# Phylogeography, taxonomy, and conservation of the endangered brown howler monkey, *Alouatta guariba* (Primates, Atelidae), of the Atlantic Forest

**DOI:** 10.3389/fgene.2024.1453005

**Published:** 2024-12-03

**Authors:** Luciana I. Oklander, Gabriela P. Fernández, Stela Machado, Mariela Caputo, Zelinda M. B. Hirano, Anthony B. Rylands, Leonardo G. Neves, Sérgio L. Mendes, Luciana G. Pacca, Fabiano R. de Melo, Italo Mourthé, Thales R. O. Freitas, Daniel Corach, Leandro Jerusalinsky, Sandro L. Bonatto

**Affiliations:** ^1^ Instituto de Biología Subtropical (IBS), Consejo Nacional de Investigaciones Científicas y Técnicas (CONICET), Universidad Nacional de Misiones (UNAM), Posadas, Argentina; ^2^ Neotropical Primate Conservation Argentina, Puerto Iguazú, Misiones, Argentina; ^3^ Primate Specialist Group, Species Survival Commission, International Union for the Conservation of Nature IUCN, Austin, TX, United States; ^4^ Centro de Bioinvestigaciones (CeBio), Universidad Nacional del Noroeste de la Provincia de Buenos Aires (UNNOBA), Centro de Investigaciones y Transferencia del Noroeste de la Provincia de Buenos Aires (UNNOBA-UNSAdA-CONICET), Buenos Aires, Argentina; ^5^ Programa de Pós-Graduação em Ecologia e Evolução da Biodiversidade, Pontifícia Universidade Católica do Rio Grande do Sul, Porto Alegre, Rio Grande do Sul, Brazil; ^6^ Facultad de Farmacia y Bioquímica, Departamento de Microbiología, Inmunología, Biotecnología y Genética, Cátedra de Genética Forense y Servicio de Huellas Digitales Genéticas, Universidad de Buenos Aires-CONICET, Buenos Aires, Argentina; ^7^ Projeto Bugio, Centro de Pesquisas Biológicas de Indaial—CEPESBI, Universidade Regional de Blumenau—FURB, Indaial, Santa Catarina, Brazil; ^8^ Re:wild, Austin, TX, United States; ^9^ Independent Researcher, Ilhéus, Brazil; ^10^ Instituto Nacional da Mata Atlântica—INMA, Ministério da Ciência, Tecnologia e Inovação—MCTI, Santa Teresa, Espírito Santo, Brazil; ^11^ Centro Nacional de Pesquisa e Conservação de Primatas Brasileiros—CPB, Instituto Chico Mendes de Conservação da Biodiversidade—ICMBio, Cabedelo, Brazil; ^12^ Departamento de Engenharia Florestal, Universidade Federal de Viçosa, Viçosa, Brazil; ^13^ Departamento de Genética, Universidade Federal do Rio Grande do Sul, Porto Alegre, Rio Grande do Sul, Brazil; ^14^ Programa de Pós Graduação em Genética e Biologia Molecular, Universidade Federal do Rio Grande do Sul, Porto Alegre, Rio Grande do Sul, Brazil; ^15^ Programa Macacos Urbanos, Universidade Federal do Rio Grande do Sul, Porto Alegre, Rio Grande do Sul, Brazil; ^16^ Escola de Ciências da Saúde e da Vida, Pontifícia Universidade Católica do Rio Grande do Sul, Porto Alegre, Rio Grande do Sul, Brazil

**Keywords:** Platyrrhini, primates, microsatellite, mitochondrial DNA, threatened species

## Abstract

The brown howler, *Alouatta guariba*, endemic to the Atlantic Forest of Brazil and Argentina, is threatened by habitat loss and fragmentation, hunting, and its susceptibility to yellow fever. Two subspecies have been recognized, but their names, validity, and geographic ranges have been controversial. We obtained samples covering the species' entire distribution in Brazil and Argentina to clarify these issues by investigating their genetic diversity and structure and assessing their evolutionary history. We analyzed, for the first time, a set of ten microsatellite markers (N = 153), plus mitochondrial DNA (mtDNA) segments of the control region (N = 207) and cytochrome b gene (N = 116). The microsatellite data support two to three genetic clusters with biological significance. The southern populations (Argentina, Santa Catarina, and Rio Grande do Sul) presented a homogeneous genetic component, and populations from São Paulo (SP) to the north presented another component, although most presented ∼20% of the southern component. With K = 3, SP emerged as a third component while sharing some ancestry with Rio de Janeiro and Argentina. The mtDNA phylogenies revealed three main clades that diverged almost simultaneously around 250 thousand years ago (kya). Clades A and B are from central SP to the north and east, while clade C is from SP to the south and southwest. Samples from SP presented haplotypes in all three clades, sometimes in the same population. The demographic history of the species estimated with the Bayesian skyline plot of the mtDNA showed a strong expansion ∼40–20 kya and a strong reduction over the last ∼4–2 kya. Although the genetic clusters identified here deserve appropriate management strategies as conservation units, the absence of (i) concordance between the mtDNA and microsatellite data, (ii) reciprocal monophyly in the mtDNA, and (iii) clear-cut non-genetic diagnostic characters advises against considering them as different taxonomic entities. None of the previous taxonomic proposals were corroborated by our data. Our results elucidate the taxonomy of the Atlantic Forest brown howler, indicating it should be considered a monotypic species, *A. guariba*. We also clarify the evolutionary history of the species regarding its intraspecific genetic diversity, which is crucial information for its conservation and population management.

## 1 Introduction

**FIGURE 1:**
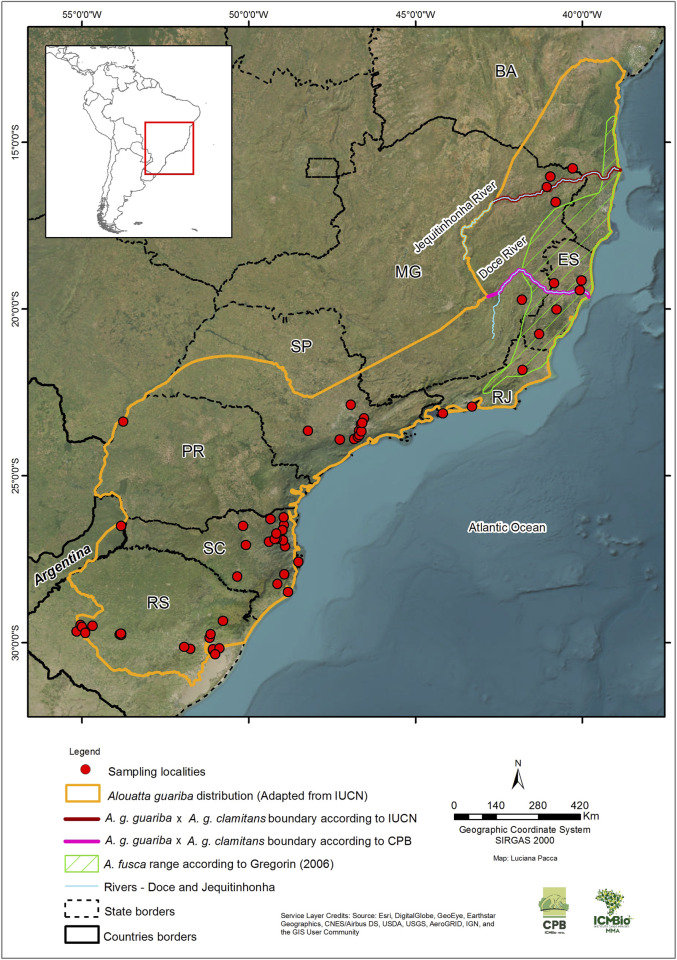
Sites (N = 65) where 223 brown howler monkey samples (*Alouatta guariba*) were collected in eastern Brazil and northeastern Argentina for the present study. We know only the state of origin for 20 of the samples, so the collecting sites are not shown on the map. The species distribution range was adapted from the IUCN Red List of Threatened Species (Jerusalinsky et al., 2021). The boundaries by the IUCN Red List (Rio Jequitinhonha) and CPB/ICMBio (Rio Doce) for *Alouatta guariba guariba* (Neves et al., 2018; 2021) and *Alouatta guariba clamitans* (Bicca-Marques et al., 2018; Buss et al., 2021) correspond to those proposed by Kinzey (1982) and Rylands et al. (1988), respectively. The range of *Alouatta fusca* is according to Gregorin [2006—adapted from Povill et al. (2023)]. Brazilian State abbreviations: BA, Bahia; MG, Minas Gerais; ES, Espírito Santo; RJ, Rio de Janeiro; SP, São Paulo; PR, Paraná; SC, Santa Catarina; RS, Rio Grande do Sul.

The brown howler monkey, *Alouatta guariba* (É. Geoffroy Saint-Hilaire in Humboldt & Bonpland, 1811: *p*.355), is endemic to the Atlantic Forest of Brazil and Argentina. It occurs in eight states along eastern, southeastern, and southern Brazil, in the states of Bahia, Espírito Santo, Minas Gerais, Rio de Janeiro, São Paulo, Paraná, Santa Catarina, and Rio Grande do Sul, and in the Argentinian province of Misiones (Jerusalinsky et al., 2021). The type locality provided by Humboldt was merely “Brazil,” but Cabrera (1957) restricted it to the “Rio Paraguaçú [just north of the state capital, Salvador] in the state of Bahia, Brazil” (Figure 1), indicating a geographic distribution that included the Atlantic Forest of the states of Bahia, Espírito Santo, and Minas Gerais. Gregorin (2006) rightly pointed out that Cabrera’s restriction was not based on any reference specimen and should, as such, be considered invalid.

The species was once referred to as *Alouatta fusca*, named *Stentor fuscus* É. Geoffroy Saint-Hilaire, 1812: *p*.108. Hershkovitz (1963) believed that the name “*guariba*”, mentioned by Geoffroy Saint-Hilaire (1806), was a junior synonym of the red-handed howler, *Alouatta belzebul* (Linnaeus, 1766), which would thus be a senior homonym of *Simia guariba* listed by Humboldt (1811), disallowing the use of “*guariba*” for naming another member of the genus. Rylands and Brandon-Jones (1998) argued that Geoffroy Saint-Hilaire (1806) wrote it only as a vernacular name, not as a binomial. It was not, as such, a homonym, and Humboldt’s *S. guariba*, attributed to Geoffroy Saint Hilaire and published two months before *A. fusca* (cf. Thomas, 1913), was as such valid. Gregorin (2006), however, in his exhaustive and detailed review of the Brazilian howlers, re-affirmed the stance of Hershkovitz in considering that “*guariba*” was indeed a junior synonym of *A. belzebul* and maintained *A. fusca*, emphasizing the confusion of Humboldt’s 1811 treatment of the howler monkey taxonomy. For the present, at least, *A. guariba* has prevailed over *A. fusca*, following Cabrera (1957), Hill (1962), Rylands and Brandon-Jones (1998) and Groves (2001; 2005).

A subspecies was described in 1940: *A. guariba clamitans* Cabrera, 1940: *p*.404. Its type locality was given as “Alto da Serra, São Paulo,” which was imprecise. Cabrera (1957) subsequently restricted it to the “Serra da Cantareira, São Paulo” merely, however, on the basis of its abundance there, as had been indicated by Vieira (1944) (Figure 1). Gregorin (2006) provided a more precise type locality based on a lectotype—“São Sebastião, [on the coast of the] state of São Paulo” (Figure 1). Cabrera (1957) indicated a geographic distribution for this subspecies encompassing the states of Rio de Janeiro, São Paulo, Paraná, Santa Catarina, and Rio Grande do Sul, extending into the Atlantic Forest of the province of Misiones in Argentina, implying that the nominate form was restricted to the states of Espírito Santo, eastern Minas Gerais and Bahia, south of the Rio Paraguaçú. As such, the two subspecies were named the northern brown howler (*A. g. guariba*) and the southern brown howler (*A. g. clamitans*). To avoid confusion, from here on, we will refer to both as subspecies and Gregorin’s *A. fusca* (see below) as *A. g. guariba.*

The taxonomy and the geographic distributions of the two named subspecies have been, however, to this day, subjects of much debate, with the differing nomenclatures and no understanding of a precise or even fuzzy delimitation of their ranges, despite much prospection based on pelage color, cytogenetic analysis, and molecular genetic studies based on mitochondrial DNA (mtDNA). Gregorin’s (2006) taxonomic review was based on pelage coloration and cranial and hyoid morphology. The variation in pelage in the brown howlers is considerable, and Gregorin concluded on this basis that the two brown howlers were distinct species: the southern *A. g. clamitans* he found to be sexually dichromatic—the female species darker than the male species—whereas the two sexes are similar in the northern *A. g. guariba*. The pelage color and patterns, however, are arguably insufficient to support their validity because they change across the species distribution, apparently according to a latitudinal gradient, with male species being lighter in southern regions and darkening to the north while, although less intense, the inverse process happens in female species. Some populations do not present sexual dichromatism (Gregorin, 2006). Remarkably, the color of their coats results from the reddish-colored secretion produced by epidermal glands, which is related to the level of testosterone and iron in the bloodstream (Hirano et al., 2003; Hirano, 2004). Thus, coat color may change throughout the species distribution, as the release of secretion may vary according to seasonal changes and environmental factors, such as the type of food available, weakening the pelage coloration patterns as a diagnostic character. Prior to Gregorin’s study, *A. g. clamitans* was considered to be the form in the southern and central part of the species’ distribution, and *A. g. guariba* was the form found in a more restricted area to the north, but the boundary between the two was undecided. Kinzey (1982) indicated that it was the Rio Doce in Minas Gerais and Espírito Santo, whereas Rylands et al. (1988) found evidence that it was the Rio Jequitinhonha, further north in Minas Gerais and Bahia. Gregorin (2006) identified a slightly different pattern, with *A. g. guariba* (his *A. fusca*) being largely restricted to the coastal areas in southern Bahia, north of the Rio Jequitinhonha and south of the lower Jequitinhonha into Espírito Santo, extending west somewhat to the middle Rio Doce basin in Minas Gerais. The southernmost locality he recorded was Teresópolis, in the state of Rio de Janeiro. In its Brazilian range, Gregorin recorded *A. g. clamitans* east of the Uruguay and Paraná rivers in Rio Grande do Sul, Santa Catarina, Paraná, and São Paulo and in coastal Rio de Janeiro. Populations further north were identified from the upper Rio Doce and on both the northern and southern banks of the middle to upper Rio Jequitinhonha, both in Minas Gerais. The pattern identified for the Rio Jequitinhonha valley conferred with observations made by Rylands et al. (1988). A genetic study by Povill et al. (2023) indicated three clades, two of the northern subspecies, *A. g. guariba*, and one of the southern subspecies, *A. g. clamitans*, and used ecological niche-modeling to predict their distributions. Their findings indicated that *A. g. guariba* occurs further south than had been previously recognized, extending well south of the Rio Jequitinhonha through Espírito Santo into eastern Rio de Janeiro, whereas *A. g. clamitans* extended north, inland from *A. g. guariba* from Rio de Janeiro to the Jequitinhonha valley in northeastern Minas Gerais—a proposal very similar to that of Gregorin (2006).

The species is threatened by habitat loss and fragmentation, hunting, trafficking for the pet trade, roadkill, electrocution, and dog attacks (Jerusalinsky et al., 2021; Chaves et al., 2022) and is especially susceptible to the yellow fever virus, with recent outbreaks resulting in the decimation of many of its populations (Almeida et al., 2012; Moreno et al., 2015). The synergistic effects of these threats have resulted in reduced and isolated populations and local extinctions throughout its distribution (Jerusalinsky et al., 2021; Oklander et al., 2022; 2024). These declines have resulted in its listing among the world’s most endangered primates (Oklander et al., 2022). It is currently categorized as Vulnerable on the IUCN Red List of Threatened Species (Jerusalinsky et al., 2021) due to its relatively large extent of occurrence with a recommendation that it should be elevated to Endangered.

Strategies for the species’ conservation have been defined as official public policies in Brazil and Argentina through national action plans (ICMBio, 2018; MAyDS, 2021), including express population management approaches (Oklander et al., 2024). However, the effective implementation of such strategies is hindered by the species’ inconclusive taxonomy—the existence (or not) of two distinct taxa and their geographic limits. More comprehensive molecular data and more representative sampling are decisive for this.

Phylogeographic studies allow us to understand the impact that paleogeoclimatic events have had on the species through the analysis of the present distribution and demographic history imprinted in the genomes of the individuals. In the interglacial periods of the Quaternary, the extent of the Atlantic Forest was evidently similar (if more widespread) to the present, but the forest contracted during glacial periods, and the region was dominated by grassland (Câmara, 1991; Behling and Negrelle, 2001; Behling et al., 2005; Carnaval and Moritz, 2008; Pessenda et al., 2009; Woodburne, 2010). These changes would, of course, broadly affect the distribution and populations of arboreal mammals (Lister, 2004). Kinzey (1982) and Rylands et al. (1996) discussed what has been referred to as Pleistocene Forest refuges and, based on primates, identified three centers of endemism in the Atlantic Forest—the Bahia Center, the Rio Doce Center, and the Paulista Center—that would be pertinent to the phylogeographic history and evolution of the brown howlers. These centers are largely montane, and the refugees would be dendritic and complex, depending on soil, altitude, and local climates, but there is no doubt they would have been forces for speciation, affecting the genetic makeup of populations in the range of the howler monkeys today.

In this study, we analyzed, for the first time for this species, a set of microsatellite markers of the nuclear DNA, in combination with segments of the control region and the cytochrome b gene of the mtDNA. Our objective was to investigate the genetic diversity, genetic structure, and demographic history of *A. guariba* throughout its distribution and discuss our findings in a taxonomic and conservation context. Finally, we propose three management units (MU) to support protection and management strategies for the species.

## 2 Material and methods

### 2.1 Sample collection

We obtained samples of 243 individuals of *A. guariba* from 65 localities in 51 municipalities in the eight Brazilian states (from north to south: Bahia, BA; Minas Gerais, MG; Espírito Santo, ES; Rio de Janeiro, RJ; São Paulo, SP; Paraná, PR; Santa Catarina, SC; and Rio Grande do Sul, RS) and the Argentinian (ARG) province of Misiones along the species range (Figure 1; Table 1; Supplementary Material 1). Most of the samples (172) were from individuals of precisely known origin collected in the field. The geographic coordinates for these were recorded with GPS equipment or later attributed to the location or vicinity of the individuals’ origin (for which we consider a margin of error of up to 10 km). For 51 individuals sampled, however, we had information only on their municipality of origin but not the precise site. The geographic coordinates for these were assigned to the centroid of the municipality or protected area of origin, assuming a margin of error of up to 50 km in relation to the precise origin. For the other 20 samples, we had information only about the state in which they were collected, with no details about the site or even the municipality of origin. Although they were assigned the coordinates of the centroid of the species distribution in the state where the individuals were sampled—and for which the margin of error could be more than 100 km—, these individuals were not included in the analyses that used geographical location.

**TABLE 1:** Sample localities by country and state/province, with the corresponding geographical coordinates and the number of samples per site.

Country, State/Province	Sampling site/municipality	Geographic coordinates (lat, long)		Number of samples
Brazil, Bahia	Pouso Alegre	−15,786,122	−40,293,220	1
Brazil, Minas Gerais	Fazenda Santa Rosa, Pedra Azul	−16,030,122	−40,955,918	7
	Jequitinhonha	−16,348,105	−41,059,708	1
	Felisburgo	−16,793,299	−40,797,148	4
	RPPN F. M. Abdala, Caratinga	−19,723,208	−41,806,262	9
Brazil, Espírito Santo	Sooretama	−19,141,640	−40,026,768	3
	Pancas	−19,229,909	−40,850,546	7
	Linhares	−19,443,691	−40,074,337	2
	Santa Maria de Jetibá	−20,017,945	−40,772,516	7
	Floresta Nacional de Pacotuba	−20,745,503	−41,292,414	11
Brazil, Rio de Janeiro	Parque Estadual do Desengano	−21,823,500	−41,794,000	1
	Rio de Janeiro	−22,927,162	−43,313,209	2
	Ilha Grande, Angra dos Reis	−23,130,444	−44,182,000	1
Brazil, São Paulo	Campinas	−22,869,818	−46,940,746	3
	Mairiporã	−23,280,865	−46,539,754	5
	Serra da Cantareira	−23,415,583	−46,601,663	19
	Horto Florestal, São Paulo	−23,452,453	−46,633,679	3
	Bairro Santo Amaro, São Paulo	−23,649,109	−46,692,290	1
	Itapetininga	−23,651,029	−48,224,630	1
	PE Fontes do Ipiranga, São Paulo	−23,670,784	−46,628,888	4
	Jardim Varginha, São Paulo	−23,789,855	−46,686,745	1
	Parelheiro	−23,831,775	−46,732,522	4
	Embu-Guaçu	−23,898,365	−46,836,205	1
	Ibiúna	−23,910,732	−47,269,401	1
Brazil, Paraná	Mata do Bugio, Alto Paraíso	−23,380,339	−53,767,517	9
Brazil, Santa Catarina	Joinville	−26,244,200	−48,951,400	1
	São Bento do Sul	−26,295,500	−49,350,600	3
	Guaramirim	−26,476,200	−48,945,200	1
	Papanduva	−26,498,700	−50,171,400	1
	Massaranduba	−26,626,400	−48,988,300	1
	Pomerode	−26,728,600	−49,173,300	9
	Vila Itoupava, Blumenau	−26,729,108	−49,060,589	3
	Itoupavazinha, Blumenau	−26,846,891	−49,109,757	8
	Morro Geisler, Indaial	−26,898,227	−49,221,323	11
	Encano Baixo, Indaial	−26,898,331	−49,164,649	1
	Gaspar	−26,931,000	−48,965,800	1
	Ascurra	−26,973,400	−49,395,600	2
	Taio	−27,078,700	−50,092,700	1
	Brusque	−27,124,700	−48,909,700	1
	Florianópolis	−27,577,900	−48,508,200	2
	São Bonifácio	−27,956,200	−48,939,900	1
	Lages	−28,020,100	−50,340,900	2
	Braço do Norte	−28,240,900	−49,142,800	1
	Laguna	−28,486,000	−48,826,000	3
Brazil, Rio Grande do Sul	Canela	−29,351,900	−50,774,900	1
	Perseverança, São Francisco de Assis	−29,470,133	−55,045,017	1
	Balneário, Jaguari	−29,493,531	−54,689,881	5
	Reserva, São Francisco de Assis	−29,527,724	−55,000,199	2
	Cerro Negro, São Francisco de Assis	−29,613,519	−54,970,608	3
	Jacaquá, São Francisco de Assis	−29,664,096	−55,166,062	1
	Cerro Loreto, São Vicente do Sul	−29,710,571	−54,901,771	11
	Cerro Agudo, São Vicente do Sul	−29,712,242	−54,903,007	2
	Gravatá 2, Santa Maria	−29,735,697	−53,828,831	2
	Sede, Santa Maria	−29,738,332	−53,843,033	1
	São Leopoldo	−29,755,400	−51,145,000	1
	Capão da Infantaria, Santa Maria	−29,757,532	−53,877,324	3
	Piquenique, Santa Maria	−29,778,939	−53,830,357	3
	Esteio	−29,851,700	−51,176,800	1
	RS290 km161, Butiá	−30,132,827	−51,938,269	1
	Águas Claras, Viamão	−30,173,690	−50,882,029	2
	Arroio dos Ratos	−30,193,200	−51,751,900	1
	Morro Extrema, Porto Alegre	−30,200,521	−51,075,412	1
	Lami, Porto Alegre	−30,248,088	−51,067,817	8
	Itapuã, Viamão	−30,354,037	−51,010,523	7
Argentina, Misiones	Parque Provincial Piñalito	−26,500,000	−53,833,333	5
TOTAL	65			223

For most analyses, we used the sample geographical locale as described above. For the graphical depiction of some results, we refer to the samples using their Brazilian state of origin. We consider this organization as appropriate, as the species is mainly distributed on a north–south axis the same way as these states, and most sampling efforts were state-centric. Hence, this organization mainly reflects the sampling units, and it is a practical way to organize the sampling sites.

Based on the geographic location of each sample, we assigned the presumed species/subspecies classification based on the previously existing taxonomic and distribution proposals by (1) IUCN [Neves et al., 2018 for *A. g. guariba*; Buss et al., 2021 for *A. g. clamitans*—which follows the proposal by Rylands et al. (1988)], (2) CPB/ICMBio [Neves et al., 2018 for *A. g. guariba*; Bicca-Marques et al., 2018 for *A. g. clamitans*—which follows the proposal by Kinzey (1982)], (3) Gregorin (2006—*A. fusca* = *A. g. guariba; A. clamitans* = *A. g. clamitans*), and (4) Povill et al. (2023—Northern clade = *A g. guariba*; Southern clade = *A. g. clamitans*) (Supplementary Material 1). According to this, our sampling included individuals from the distribution of both *A. g. guariba* (n = 9, IUCN; n = 23, CPB/ICMBio; n = 31, Gregorin, 2006; n = 61, Povill et al., 2023) and *A g. clamitans* (n = 231, IUCN; n = 217, CPB/ICMBio; n = 208, Gregorin, 2006; n = 144, Povill et al., 2023), in addition to some samples with doubtful classification following these criteria (n = 3, IUCN and CPB/ICMBio; n = 4, Gregorin, 2006; n = 38, Povill et al., 2023).

We collected three different types of samples: blood (n = 5), tissue (n = 78), and feces (n = 160) (Supplementary Material 1). For blood samples, an amount of 500 µl–1 mL of whole blood was collected in EDTA tubes or ethanol (>70%) from individuals kept at different Brazilian zoos during routine care procedures at these institutions, conducted under the technical responsibility of the zoos’ qualified teams, always including a veterinary doctor, duly authorized by the competent bodies for the supervision of *ex situ* keeping institutions. For tissue samples, an amount of ca. 1 cm³ of muscle (usually thigh, back, or abdomen) was collected in ethanol (>70%) only from dead animals, the carcasses of which were kept in authorized institutional collections or which were found during fieldwork. For the latter, it was possible to identify a variety of causes (electrocution on electricity transmission lines, run over by a vehicle, domestic dog attack, fight with a social group, and excessive parasite infection), although it was not possible to determine the cause of death in some cases. Fecal samples were collected immediately after defecation during fieldwork that was, for the most part, specifically dedicated to obtaining samples for this study. All fecal samples were preserved at 24°C in 50 mL screw-top tubes containing solid NaCl or ethanol (>70%) (Oklander et al., 2004) until DNA extraction. Only one type of sample per individual was used. Samples are in the collection of the Escola de Ciências da Saúde e da Vida, Pontifícia Universidade Católica do Rio Grande do Sul, Porto Alegre, Rio Grande do Sul, Brazil. Samples were collected in previous studies (Jerusalinsky, 2001; Machado, 2011; Mourthé et al., 2019).

No howlers were killed, captured, or sedated in the field to obtain samples for the present study. No individuals were killed, captured, sedated, or held captive in *ex situ* conditions exclusively to obtain samples for this project. All samples were collected in full compliance with the Code of Best Practices for Field Primatology (IPS & APS, 2014) and, following the Argentine and Brazilian laws, sampling was conducted with permission from the respective National Environmental Agencies (Brazil: SISBIO n. 19,927-12 by ICMBio, CONICET n. 11420110100322CO and Ministry of Ecology, Misiones province, Argentina n. 9910-00086/17). Note that Brazilian legislation establishes that the collection of samples of biological material in all the situations described above and outside protected areas does not require collection permits.

### 2.2 DNA extraction

DNA was extracted from blood or tissue samples using the standard phenol–chloroform protocol (Sambrook et al., 1989) and from feces samples using a QIAmp DNA Stool Mini Kit (QIAGEN). For DNA extracted from fecal samples, PCR and sizing were repeated twice (in the case of a heterozygous genotype call) or four times (in the case of a homozygous genotype call) to minimize possible genotyping errors due to allelic dropout (Pritchard et al., 2000; Peakall et al., 2003). We only recorded an allele if it was observed at least twice in different amplifications from the same DNA extract. All amplification assays included negative controls. Every step of the laboratory work was carried out in specific laboratory spaces (DNA extraction room and PCR room), inside laminar flow hoods with negative pressure, and using aerosol-resistant filter tips to avoid between-sample cross-contamination.

### 2.3 Microsatellite genotyping

Ten microsatellites or short tandem repeats (STRs) (AB7, AB10, AB17, AC14, AC17, D8S165, D17S804, TGMS1, 1118, and 157) (Tomer et al., 2002; Di Fiore and Fleischer, 2004; Gonçalves et al., 2004; Oklander et al., 2007a) were amplified for 153 individuals (Figure 1; Supplementary Material 1). These markers have already been characterized as polymorphic in two howler species (*Alouatta caraya* and *A. guariba*) and proven to be useful for population demographic and reproductive studies (Oklander et al., 2007a; Oklander et al., 2007b; Oklander et al., 2010; Oklander et al., 2017; Oklander et al., 2022).

Genotyping PCRs was performed using 5–10 ng of the DNA template for tissue samples or 5 μL of the extraction of fecal samples, 100 µM dNTPS, 1.8 mM MgCl2, 0.02 µM of forward primer, 0.1 µM of reverse primer, 0.4 µM of fluorescence labeling (Schuelke, 2000), 1 M betaine, 5% trehalose, 1x buffer, and 0.1 unit of Platinum Taq DNA Polymerase (Invitrogen). PCR amplification profile: denaturation at 94°C for 3 min, followed by 11–15 cycles at 94°C for 45 s, 65°C (−0.5°C/cycle) for 1 min, 72°C for 90 s and 26–30 cycles at 94°C for 45 s, 58°C–60°C for 1 min, 72°C for 90 s and finished with a final extension at 72°C for 30 min. For the AB17 locus, we used six cycles at 60°C (−1°/cycle) followed by 30 cycles at 50°C, and for the AC14 locus, we used 30 cycles at 61°C instead of the touch-down condition.

PCR products labeled with different fluorochromes were combined and co-injected with HD400-ROX as an internal size standard to be separated by electrophoresis on a MegaBACE 1000 (Pharmacia, Upsala, Sweden) automated sequencer (Amersham Biosciences) according to the manufacturer’s protocol. Alleles were manually scored by performing a visual inspection of electropherograms after developing the bin panel for each locus in Genetic Profiler 2.2 (GE Healthcare). Genotypes were screened for null-alleles and to discriminate between errors in allele frequency estimates caused by null-alleles, allele dropout, or stutter bands using Micro-Checker v2.2.332 (Van Oosterhout et al., 2004).

#### 2.3.1 Microsatellite analysis. Genetic diversity

Numbers of different alleles, effective and private alleles, observed heterozygosity (Ho), expected heterozygosity (He), and unbiased expected heterozygosity (uHe) were computed with the software GenAlEx v6.5 for each locus and population (Peakall and Smouse, 2012). Deviations from the Hardy–Weinberg equilibrium (HWE) were assessed by employing an exact test and using Arlequin v 3.5 software (Excoffier and Lischer, 2010). The F_IS_ inbreeding coefficient per population and gene diversity (Hs) was calculated for each locus in a population using Fstat software v2.9.4 (Goudet, 2003).

${HS}^{K}=\frac{n_{k}}{\begin{array}{cc} n_{k} & -1 \end{array}}\left(1-\sum p^{2}ki-{Ho}^{k}/2n_{k}\right)$ , where n represents the number of alleles, *p* represents the allele frequency, and Ho represents the observed heterozygosity.

#### 2.3.2 Structure analysis

Genetic structure was evaluated using non-spatial Bayesian clustering with the Structure v.2.3.4 program (Pritchard et al., 2000). A series of 20 independent runs per K (ranging from 1 to 7) was conducted using the admixture model with correlated allele frequencies, without prior information about sampling locations and independent allele frequencies, and with 1,000,000 Monte Carlo–Markov iterations after a burn-in of 50,000 replicates. Several K-estimation methods were tested, including the ΔK method (Evanno et al., 2005) and the parsimony method (Wang, 2019) using the KFinder software (Wang, 2019). Bar plots were constructed with the software Pophelper (Francis, 2016).

Discriminant analysis of principal components (DAPC) was employed to assess the pattern of microsatellite genetic variability among individuals, using the *dapc* function from the R package *adegenet* (Jombart and Ahmed, 2011), and we used the seven major sampling areas (the Brazilian states and Argentina, as described above at 2.1) as prior information. The number of principal components that explained 90% of the cumulative variance was retained.

We estimated pairwise F_ST_ (Weir and Cockerham, 1984) between major areas using the *hierfstat* (version 0.5-11) R package, which was used to perform a Mantel test (with the “gl.ibd” function from the *dartR* package with default options) and an isolation-by-distance (IBD) plot to test whether the geographic distances between sampling sites correlated with the genetic distances.

We complemented the population genetic structure analysis with the TESS3 approach (Caye et al., 2016) and the *tess3r* R package (http://bcm-uga.github.io/TESS3_encho_sen) using default parameters for K = 2 and K = 3, with ten repetitions for each value of K, to estimate individual ancestry coefficients and genetic groups, considering the sample geographic coordinates.

### 2.4 Mitochondrial marker sequencing

We performed amplifications and sequencing of two mitochondrial fragments. Approximately 700 base pairs (bp) of the mtDNA cytochrome b gene (CytB) were amplified with the primers L14724 (Irwin et al., 1991) and (5′-TGG​GTC​GGT​TAG​AAG​GTC​AG-3′) designed by us for this work. The second mtDNA marker analyzed was a fragment of approximately 500 bp of the control region (CR) amplified with primers How-RA1 and How-S7 (Ascunce et al., 2003).

For DNA extracted from feces, a nested PCR was performed with internal primers for CR RC_intF2 (5′-AAA​ATG​TGG​GCG​GGT​TGT-3′) and RC_intR1 (5′-CAT​AGC​ACA​TTC​GTC​CCG​TA-3′) designed by us for *A. guariba*. The PCRs contained approximately 10–100 ng of total DNA, 100–150 µM dNTPS, 1.5 mM MgCl_2_, 0.15 µM each primer, 0.1 mg/mL BSA, 1x buffer, and 0.4–1.25 units of Platinum Taq DNA Polymerase (Invitrogen). The first amplification started with an initial denaturation at 94°C for 3 min, followed by eight cycles at 94°C for 45 s, 57°C (−1°C/cycle) for 1 min, 72°C for 90 s, and 35 cycles at 94°C for 45 s, 50°C for 1 min, 72°C for 90 s and finished with a final extension at 72°C for 5 min. For amplifications with internal primers, only 30 cycles at 50°C were used. PCRs for CytB followed the same protocol as the CR, and we use internal primers only for CR.

Amplified fragments of mtDNA were sequenced using the DYEnamic ET Dye Terminator Cycle Sequencing Kit (Amersham Biosciences). Sequencing was performed in a MegaBACE 1000 automated sequencer (Amersham Biosciences) according to the manufacturer’s protocols. The sample sizes and locations for each locus are shown in Supplementary Material 1.

#### 2.4.1 Mitochondrial DNA analyses

Sequences obtained for each set of mitochondrial data, CR (596 bp), CytB (678 bp), and combined (CR + CytB) datasets were aligned using the Muscle algorithm in MEGA7 (Kumar et al., 2016). We also compiled previously gathered data on mtDNA from the species (Supplementary Material 2).

The degree of saturation was investigated by plotting proportions of transitions and transversions against the pairwise divergence between the sequences using the Xia et al. (2003) test, implemented in the DAMBE 5.2.5 program (Xia and Xie, 2001). We examined the congruence of substitution rates between each of the data sets using the partition homogeneity test (Farris et al., 1995), as implemented in PAUP* (Swofford, 2003). The number of variable sites (S), haplotypes (h), haplotype (H_d_), and nucleotide (π) diversity and mean number of pairwise differences (k) were calculated using DnaSP 6.0 (Rozas et al., 2017). We verified the possible existence of mitochondrial DNA pseudogenes in the nucleus (NUMTs) by checking the compatibility in the length of each cytb sequence obtained with those previously published and translating said sequences into proteins. Finally, their phylogenetic position in the analysis was as expected, with no sequences identified as potential NUMTs.

#### 2.4.2 Mitochondrial DNA phylogeny and divergence times

Because there is no substitution rate for the mtDNA control region (CR) and cytochrome b (CytB) for the species, we used a Bayesian inference (BI) in BEAST 2.5.2 (Bouckaert et al., 2019) to estimate the substitution rates for both fragments using as calibration points the dates estimated by Schrago (2007). The CytB rate was estimated using the unique haplotypes of *A. guariba*, two sequences of *A. caraya* generated here, and GeneBank sequences representing all families of platyrrhine species (Supplementary Material 2). Given the absence of reliable control region sequences for several platyrrhine species, the substitution rate for this segment was estimated relative to the previously calculated rate for CytB (the latter used as a rate prior) in a partitioned analysis using only *A. guariba* that presented sequences for both fragments. The BI was performed in BEAST 2.5.2 (Bouckaert et al., 2019) using the HKY + G with six categories and the Yule tree prior. We used a lognormal relaxed clock and 50 million iterations, sampling each 5,000 times. The runs were visually inspected using Tracer 1.7.1. The substitution rate for the Platyrrhini estimated with BEAST for CytB was 1.15 × 10^−8^ site/year (confidence interval 95%: 8.91 × 10^−9^–1.41 × 10^−8^) and for CR was 2.81 × 10^−7^ (confidence interval 95%: 1.46 × 10^−7^–4.4 × 10^−7^). Next, we used the above substitution rates to estimate the relationship and divergence times between the *A. guariba* mtDNA sequences in the CR plus the CytB combined dataset. The BI phylogenetic analysis was as described above, except we used the coalescent constant size as the tree prior and the inferred tree visualized in FigTree 1.4.4 (Rambaut, 2018). A second BI tree was estimated from all 117 haplotypes from CR sequences using the same parameters described above. Additionally, a CytB maximum likelihood (ML) tree was obtained in MEGA7 (Kumar et al., 2016), including our 17 CytB haplotypes and 25 *A. guariba* sequences (Povill et al., 2023) available in GenBank*,* as well as sequences of the genus *Alouatta* (*A. belzebul, A. caraya*, *A. seniculus*, *A. macconnelli*, *A. sara*, *A. pigra,* and *A. palliata*), and those of *Ateles geoffroyi* and *Ateles belzebuth* as outgroups (Supplementary Materials 2, 5).

To assess the existence of differentiated genetic groups, the CR sequence dataset was used to perform a clustering analysis with a Bayesian analysis using BAPS v6 (Corander et al., 2008) by testing the number of clusters (K) from 2 to 10, with five replicates for each K.

#### 2.4.3 Demographic history

Following the three clades obtained in the phylogenetic analysis, Tajima’s D (Tajima, 1989) and Fu’s Fs (Fu, 1997) neutrality tests were also carried out in DNASP to assess signatures of recent historical demographic events. Significant negative values of both tests are indicative of an excess of low-frequency mutations relative to expectations under the standard neutral model (strict neutrality of variants, constant population size, lack of subdivision, and gene flow). To infer historical changes in effective population size (Ne) over time, Bayesian skyline plots (BSPs; Drummond et al., 2005) were constructed with BEAST 2.5.2 (Bouckaert et al., 2019) for the whole species and separately for the three clades found in the previous analyses. Here, we used only the 207 mtDNA CR sequences. The coalescence analysis was run with the same conditions as above, except we used the BSP tree prior. Skyline reconstructions were performed using Tracer 1.7.1 (Rambaut et al., 2018), and the median and 95% credibility intervals were plotted as a time function.

## 3 Results

### 3.1 Microsatellite results

We genotyped 10 microsatellite loci for 153 samples from the entire known range of the species (Figure 1). The brown howler populations exhibited moderate levels of microsatellite diversity. The mean number of alleles (NA) was 4.06 ± 2.38; the mean observed heterozygosity (Ho) and expected heterozygosity (He) were 0.46. The lowest values were detected in RS, whereas the highest were MG and ES, and the highest number of private alleles was found in SC (Table 2). The mean unbiased expected heterozygosity (uHe) was 0.493 ± 0.275 over all populations. No significant signal of inbreeding was found for any population. All 10 loci were polymorphic in most populations, with the number of alleles per locus ranging from 1 to 12. No significant deviation of HWE was seen at any loci for any population studied (Supplementary Material 3).

**TABLE 2:** Summary estimates of diversity at ten microsatellite loci, ±standard deviation.

Population name	N	Na	Ne	Ho	He	uHe	Hs	F_IS_	Total PA
ES	7.00 ± 0.00	3.88 ± 0.64	2.88 ± 0.62	0.714 ± 0.171	0.635 ± 0.092	0.684 ± 0.099	0.682 ± 0.102	−0.048	2
MG	8.60 ± 0.84	5.00 ± 1.33	3.40 ± 1.10	0.637 ± 0.266	0.663 ± 0.155	0.705 ± 0.166	0.710 ± 0.167	0.104	7
RJ	3.00 ± 0.00	2.30 ± 0.82	1.99 ± 0.77	0.433 ± 0.274	0.428 ± 0.216	0.513 ± 0.259	0.533 ± 0.267	0.188	2
SP	29.30 ± 2.06	6.30 ± 3.13	3.32 ± 1.53	0.560 ± 0.246	0.611 ± 0.236	0.622 ± 0.240	0.623 ± 0.240	0.100	10
SC	58.80 ± 1.34	5.40 ± 3.13	1.66 ± 0.82	0.291 ± 0.224	0.308 ± 0.228	0.311 ± 0.230	0.311 ± 0.230	0.064	14
ARG	5.00 ± 0.00	2.30 ± 0.95	1.94 ± 0.80	0.440 ± 0.363	0.398 ± 0.254	0.442 ± 0.283	0.443 ± 0.278	0.006	0
RS	34.90 ± 4.15	3.20 ± 1.62	1.37 ± 0.51	0.185 ± 0.163	0.208 ± 0.195	0.211 ± 0.199	0.212 ± 0.199	0.127	3
K = 2									
ES MG RJ SP	12.24 ± 10.60	4.39 ± 2.32	2.90 ± 1.19	0.579 ± 0.258	0.582 ± 0.203	0.628 ± 0.212	0.635 ± 0.212		21
SC ARG RS	32.90 ± 22.52	3.63 ± 2.43	1.66 ± 0.74	0.305 ± 0.276	0.305 ± 0.233	0.321 ± 0.250	0.322 ± 0.249		17
K = 3									
ES MG RJ	6.14 ± 2.52	3.71 ± 1.51	2.75 ± 1.03	0.586 ± 0.266	0.571 ± 0.194	0.628 ± 0.212	0.635 ± 0.212		11
SP	29.30 ± 2.06	6.30 ± 3.13	3.32 ± 1.53	0.560 ± 0.246	0.611 ± 0.236	0.622 ± 0.240	0.623 ± 0.240		10
SC ARG RS	32.90 ± 22.52	3.63 ± 2.43	1.66 ± 0.74	0.305 ± 0.276	0.305 ± 0.233	0.321 ± 0.250	0.322 ± 0.249		17

ES, Espírito Santo; MG, Minas Gerais; RJ, Rio de Janeiro; SP, São Paulo; SC, Santa Catarina; RS, Rio Grande do Sul; ARG, Argentine population. Na = Number of different alleles; Ne = Number of effective alleles [calculated as 1/∑ (allele frequency)^2^]; He = expected heterozygosity = 1 − ∑ (allele frequency)^2^; uHe = unbiased expected heterozygosity = [2N/(2N − 1)] * He; (Hs): Gene diversity; F_IS_ (inbreeding coefficient); PA = No of alleles unique to a single population. See Supplementary Material 3 for missing data.

**FIGURE 2:**
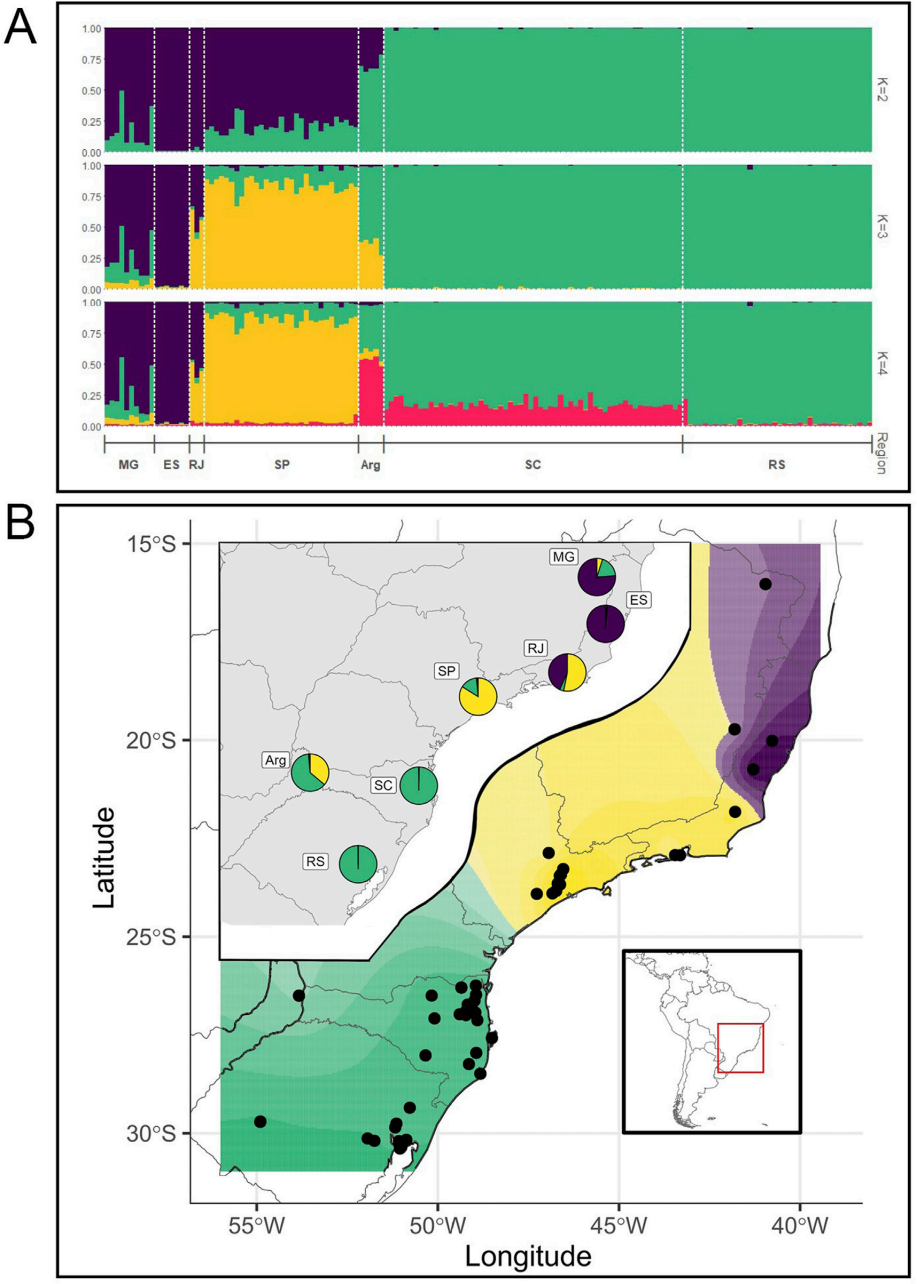
**(A)** STR genetic structure in brown howler monkeys. Membership bar plots of brown howler monkeys (N = 154) sampled across six Brazilian states and one site in northeastern Argentina, resulting from Bayesian clustering analyses in structure based on genotypic data from 10 microsatellites. Individuals are represented by vertical lines (y-axis) broken into color segments proportional to their membership coefficients to each cluster (K) and grouped into sampling locations, separated with a white dashed line. Equally colored segments share genetic ancestry and are differentiated from the others. Espírito Santo (ES); Minas Gerais (MG); Rio de Janeiro (RJ); São Paulo (SP); Santa Catarina (SC); Rio Grande do Sul (RS); and the Argentine population (ARG). **(B)** TESS spatial interpolation of the STR diversity (dots represent the individuals) assuming K = 3. Inset maps represent the proportion of each genetic cluster in each major area. Symbols as in Figure 1.

Population genetic structure based on STRUCTURE using the KFinder software for best K identified between two to three main genetic clusters (K) depending on the method. The Evanno method results showed K = 2, suggesting the existence of two major genetic groups in the species, one present mostly in individuals from the central and northern areas (SP, RJ, ES, and MG, represented in purple in Figure 2; K = 2) and the other mostly present in individuals from the southern part of the distribution (SC, ARG, and RS represented in green in Figure 2; K = 2). However, individuals from SP and MG presented a minor proportion of the southern component, while those from ARG presented a minor proportion of the central–northern component Figure 2A; K = 2).

The parsimony method supported K = 3, in which a third component (yellow in Figure 2; K = 3) is found mostly in the individuals from the central areas in SP and RJ and, to a minor degree, in Argentina. These results were consistent when performing the analyses with only eight loci. Interestingly, with K = 4, the fourth genetic component is found mainly in ARG and in a small proportion in SC, both at similar latitudes (Figure 2, K = 4).

**FIGURE 3:**
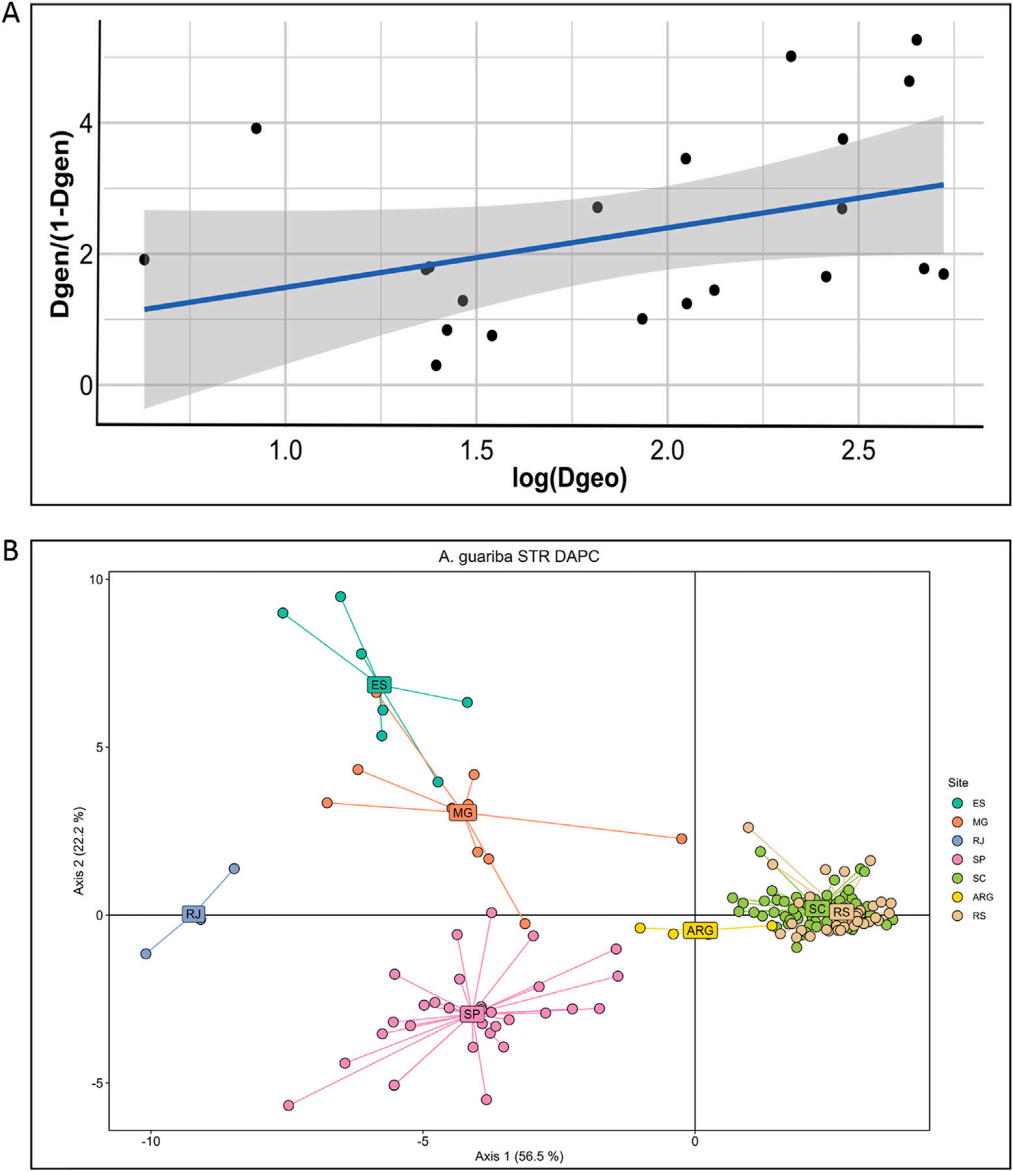
**(A)** Isolation by distance (IBD) analysis using the F_ST_ microsatellite distance (Dgeo) and the seven major geographic areas. Correlation is significant (Mantel statistic r = 0.38, *p* < 0.05). **(B)** DAPC plot of the individuals showing the first two principal axes and their proportion of variation explained. Individuals are colored according to the seven regions of origin, as in Figure 1, and labels are positioned at the centroids.

We used TESS to estimate and spatially interpolate the genetic structure. With K = 3 genetic components (Figure 2B), the geographic structure of the nuclear microsatellite variation is very clear, but again, this geographic structure is not perfect (Figure 2B, inset), as was also found with K = 2 (Supplementary Material 4). Note that TESS graphics with K = 2 show that the boundary between the main ancestry components is located at the major sampling gap between the southern and central areas (specifically between SC and SP), so part of the genetic structure may be attributed to isolation by distance. Indeed, the Mantel test between genetic and geographic distances between major areas shows a significant, although not high, correlation (r = 0.38, *p* < 0.05), as can also be seen in the IBD plot (Figure 3A).

The results of the DAPC analysis (Figure 3B) were very similar to the STRUCTURE and TESS results. Axis 1 separates individuals from the southernmost areas (RS and SC), which are tightly clustered from the central and northern populations that are more widely dispersed, with ARG individuals in an intermediate position, mirroring the K = 2 results. Axis 2 separates individuals of the central SP area from the northern populations, with RJ in an intermediate position, but again, these clusters are more dispersed, suggesting some mixed ancestry, mirroring the K = 3 results. The principal component analysis (Supplementary Material 4) results were also very similar to those of the DAPC.

When we compared the variability values (number of alleles, heterozygosity, and private alleles) between clusters, they were always higher for the MG, ES, RJ, and SP clusters (Table 1) at K = 2. In the case of K = 3, the heterozygosity values were greater for the northern cluster (MG, ES, and RJ); however, the number of alleles was greater for SP and the number of private alleles was greater in the southern cluster (ARG, SC, and RS).

### 3.2 Mitochondrial DNA results

We obtained 207 sequences of the CR and 116 of the CytB gene of *A. guariba*. The 678 bp CytB sequence alignment contained 23 variable sites, comprising 17 haplotypes, and the CR fragment of 596 bp alignment presented 147 variable sites and 117 haplotypes. Furthermore, 85 haplotypes were obtained for the combined dataset (109 sequences; 1274 pb) (Supplementary Material 2). The haplotypes generated for each locus were deposited in GenBank under accession numbers PQ278407–PQ278423 for the CytB sequences and PQ278424–PQ278540 for the CR sequences (Supplementary Material 2).

The genetic diversity indices obtained were high and similar for the three sets of sequences and both for the whole species and by clade (Table 3). For the combined set of sequences, the haplotype diversity showed values between Hd = 0.967 (Clade A) and Hd = 0.992 (Clade C), and nucleotide diversity ranged between π = 0.0089 (Clade B) and π = 0.0143 (Clade A, Table 3).

**TABLE 3:** Genetic variability for *A. guariba* populations based on the combined dataset (1274 pb, N = 109 CytB plus control region fragments) mitochondrial dataset for each operational population sampled (Pop), and by mitochondrial clade (A, B, C).

Pop–CLADES	N	S	hap	Hd	π	k	Fu-*F*s	Tajima’s *D*
Espírito Santo (ES)	8	16	6	0.929	0.0043	5.42	−0.3	−0.61
Minas Gerais (MG)	1	—	1	—	—	—	—	—
São Paulo (SP)	12	46	8	0.924	0.0153	19.48	2.586	1.28
CLADE A	21	57	15	0.967	0.01429	18.19	−0.17	0.59
Rio de Janeiro (RJ)	2	12	2	1	0.0094	12	2.49	—
Minas Gerais (MG)	2	20	2	1	0.0157	20	2.996	—
São Paulo (SP)	8	28	7	0.964	0.0074	9.43	−0.57	−0.67
CLADE B	12	40	11	0.985	0.0089	11.36	−2.477	−0.64
São Paulo (SP)	14	39	11	0.956	0.0077	9.890	−1.454	−0.84
Santa Catarina (SC)	52	92	40	0.987	0.0105	13.41	−17.39*	−1.20
Rio Grande do Sul (RS)	10	25	9	0.978	0.0072	9.18	−1.84	0.18
CLADE C	76	111	59	0.992	0.0107	13.56	−37.304**	−1.36
Overall pop	109	174	85	0.995	0.3	36.65	−27.6*	0.36

n, number of individuals sequenced; S, number of segregating sites; N_H_, number of haplotypes; Hd, haplotype diversity; π, nucleotide diversity; *SD*, standard deviation; *k*, mean number of pairwise differences. *p < 0.05.

**FIGURE 4:**
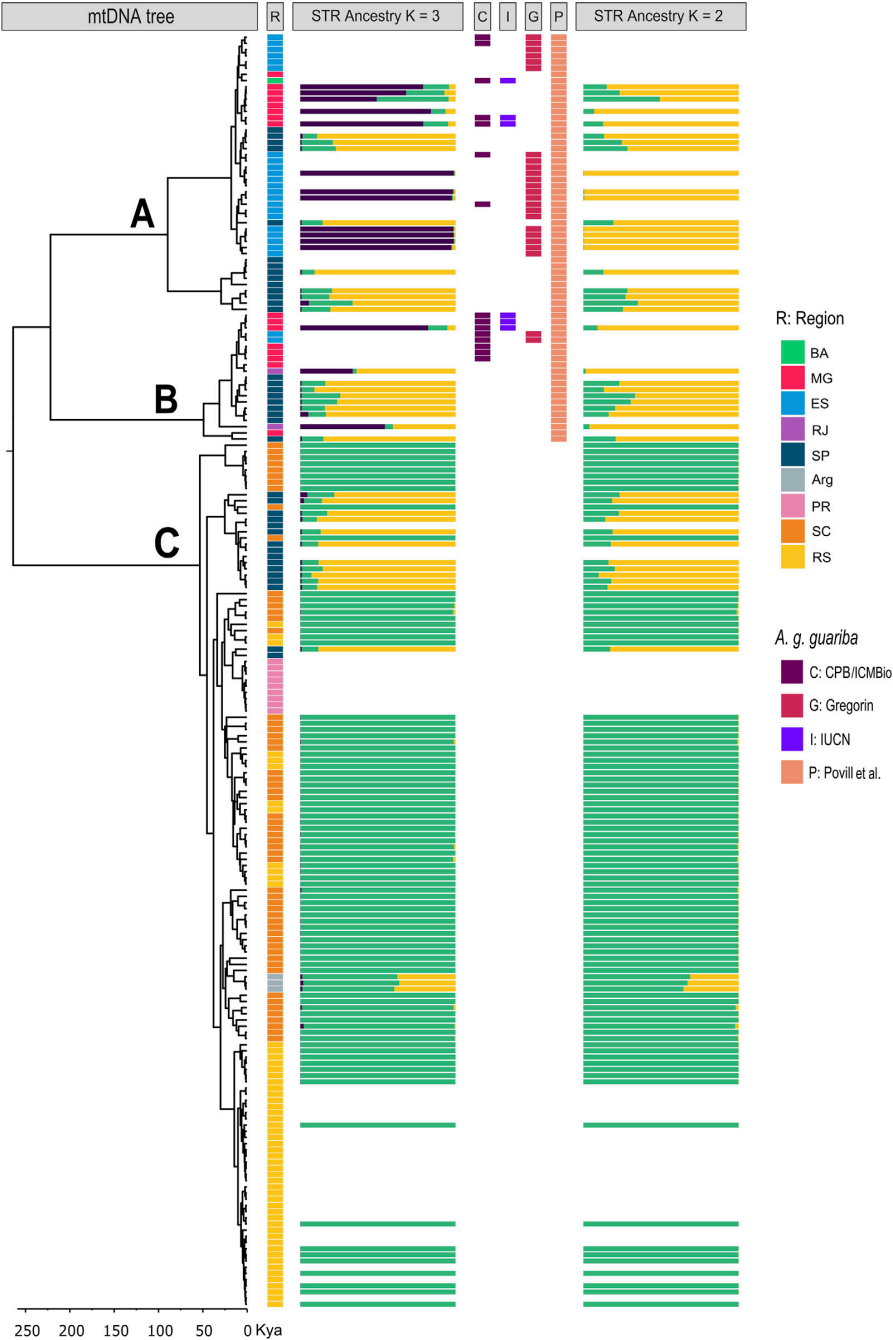
Synthesis of our results. mtDNA tree is the Bayesian phylogenetic tree with divergence times using the CR mtDNA set of sequences (N = 207 seq; 596 pb). Clade labels **(A–C)** are indicated above the branches. The bottom rule is divergence time in years. STR ancestry K = 2 and K = 3 are the results of STRUCTURE. R depicts the geographic region of the sample. C, I, G, and P depict the individual classification as *A. g. guariba* by different authors (see text for explanation).

**FIGURE 5:**
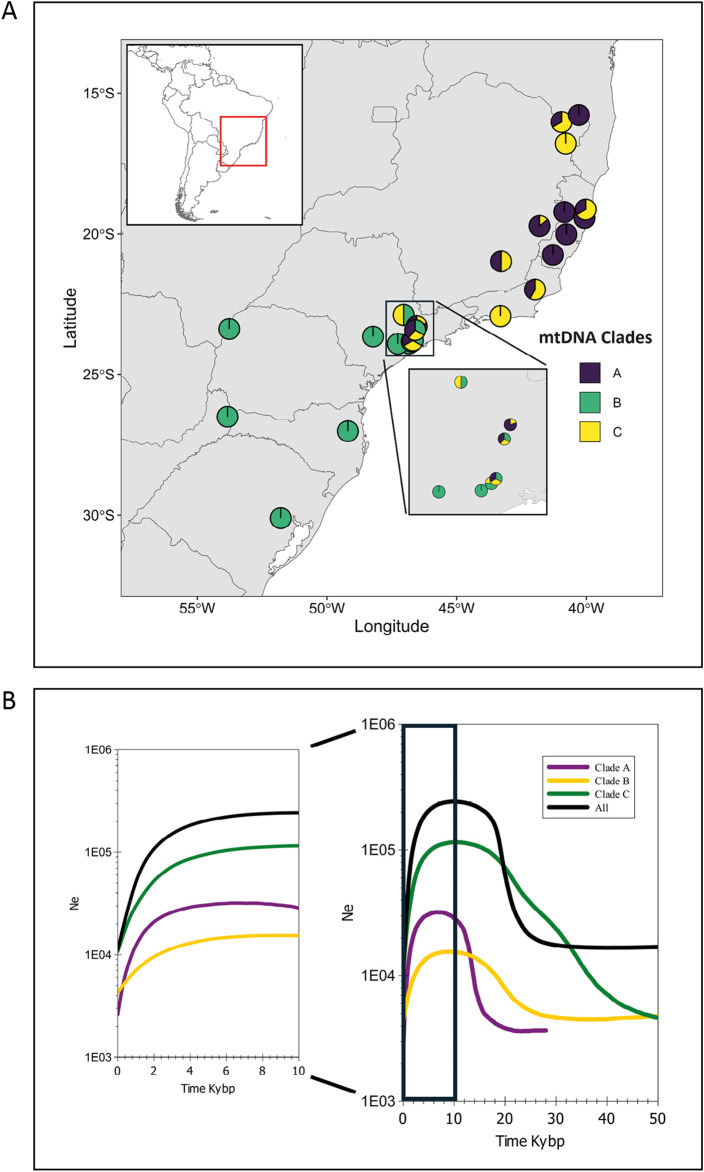
**(A)** Frequency distribution of the mtDNA control region clades (A, B, and C) by main locality and state. For Rio de Janeiro state, the upper pie chart was based on data from Povill et al. (2023). **(B)** Bayesian skyline plot for the CR set of mtDNA sequences, separated by clade and for the whole species (All), showing the effective population size (Ne) fluctuation in the last 50,000 years (kybp) and in detail for the last 10,000 years.

The three phylogenetic trees (CR, CytB, and combined dataset) found three strongly supported divergent clades (A, B, and C; posterior probability of 1; Figure 4; Supplementary Material 5). Clades A and B have individuals from the northern and central parts of the distribution, and clade C has individuals from the south and central parts (Figures 4, 5A). Clade A contains individuals from BA, ES, MG, and SP, while clade B groups contain individuals from RJ, SP, ES, and MG. ES, MG, and RJ (for CytB, Supplementary Material 5), therefore, present haplotypes from two clades (A and B), and the São Paulo area presents haplotypes from all three clades (Figures 4, 5; Supplementary Material 5). Within these clades, however, we found several subclades that are geographically structured. Notably, in clade A, there is a very divergent subclade of the SP individuals, while in clade C, there is a large clade of haplotypes from the southernmost area (PR, RS, and northern Argentina) and also from SP. The relationships between the clades A, B, and C are not resolved, as the dataset resulted in different, poorly supported topologies (Supplementary Material 5).

The Bayesian analysis of population genetic clustering (BAPS) showed six main genetic clusters along the geographic distribution of the species—three of them south of SP (one exclusive to clade C), two north of SP, and all but one present in the state of SP [Log marginal likelihood = −4,269.902; pb (K = 6) = 0.999; Supplementary Material 5].

We estimated the substitution rate of CytB for the Platyrrhini with BEAST, which shows 1.15 × 10^−8^ site/year (confidence interval 95%: 8.91 × 10^−9^–1.41 × 10^−8^) and of CR as 2.81 × 10^−7^ (confidence interval 95% 1.46 × 10^−7^–4.4 × 10^−7^). The rate found for CytB was similar to that reported for other mammals (e.g., 5.6 × 10^−9^ for *Tapirus terrestris*, Thoisy et al., 2010). The control region rate was relatively higher than that of most mammals (e.g., 2.02 × 10^−8^–5.34 × 10^−8^ for *Cerdocyon thous*, Tchaicka et al., 2007) but within the range for humans and other primates (Santos et al., 2005; Endicott and Ho, 2008). Using these rates in a combined mtDNA dataset, we estimated the divergence time between the three clades to be statistically indistinguishable: between B and A + C ∼265 thousand years ago (kya) (95% confidence interval 196–345 kya) and between clade A and C ∼245 kya (95% CI 181–319 kya). On the other hand, the estimated coalescence time for each of the three clades was greater for clade A; a first diversification process would begin at approximately 120 kya (95% CI 81–169), while for clades B and C, it would begin 75 kya (95% CI 46.4–107 kya) and 70 kya (95% CI 48.4–95.9 kya), respectively.

### 3.3 Demographic history

Considering the existence of the three major mtDNA clades and the whole species, we made several analyses with the clades separated. In general, most neutrality indices were not significant (Table 3). The exception was Fs, especially for clade C, with evidence for strong demographic changes. However, the Bayesian skyline plot demographic trajectories for the whole species and for each clade were similar in shape and timing (Figure 5B). We found a population expansion starting approximately 30–20 kya, followed by a short stability and a recent reduction between ∼4 and 2 kya. Absolute values for the female effective population sizes (Nef) reached, for the whole species, a maximum of approximately 200,000 and a minimum, at the present time, of approximately 10,000.

## 4 Discussion

### 4.1 Genetic diversity and structure

We present here the first study with multiple nuclear loci from 153 individuals encompassing all the states/provinces along the species’ distribution. For 132 of these individuals, we also sequenced the mtDNA control region, and for the majority of these, we also sequenced the mtDNA CytB. Our entire CR dataset comprises 207 sequences.

Microsatellite data indicated the presence of two to three main genetic clusters spatially structured alongside a mostly north–south axis, the third cluster being the populations from the central regions (mainly SP and partially RJ and ARG). However, only the southernmost (SC and RS) and the northeastern (ES) populations showed homogeneous ancestry, while populations from the more central areas presented mixed ancestry in different degrees, including our westernmost population from Argentina (Figure 2).

As for the mtDNA data, previous studies had shown a genetic differentiation in two main (southern and northern) clades using mitochondrial CytB, the northern with two subclades (Harris et al., 2005; Povill et al., 2023), as also suggested by Jerusalinsky (2001) from CR mtDNA data and by Machado (2011) from combined CytB + CR mtDNA data. Our Bayesian mtDNA phylogenies highly supported three major clades with similar geographic structures that we named A, B, and C, following Povill et al. (2023). However, there is no support for grouping the two northern clades (A and B) in a “northern clade,” as the posterior probability support for this grouping in our full CR dataset is small (0.4) (Figure 4). Actually, our combined (CR + CytB) mtDNA dataset grouped clades B and C, although with a posterior probability of only 0.3 (Supplementary Material 5), which suggests the absence of support for the grouping of any pair between A, B, or C clades. In addition, the three mtDNA clades have no strict correlation with the geographic distribution because ES and MG present haplotypes from two clades, and populations from the central area in SP present haplotypes from the three clades (Figures 4, 5; Supplementary Material 5).

Comparing our microsatellite and mtDNA results from the same individuals, we observe only a loose correlation between the microsatellite genetic clusters, the mtDNA clades, and the geography (Figures 2, 4, 5). For example, for K = 2, samples with mtDNA from clades A or B have mostly a nuclear genetic component (purple), although with some significant admixture, and samples with mtDNA clade C that are from the southernmost areas (RS and SC) have a specific genetic component (green, Figures 2, 4, 5). However, for K = 3, samples from SP have another component (yellow) admixtured with RJ and ARG. Samples with ancestry mostly of the third component (purple) have mtDNA from clade A, although a few are from clade B (Figures 2, 4, 5).

### 4.2 Taxonomic implications

What are the implications of our findings for the taxonomy of the species? Comparing our results to the two previous taxonomic proposals for the species (Cabrera, 1957; Hill, 1962; Rylands et al., 1995; 2000; Groves, 2001; Mittermeier et al., 2013; Gregorin, 2006; Povill et al., 2023; Figures 1, 4)—more specifically to the existence of a northern taxon, *A. guariba guariba* or *A. fusca—*and the four biogeographical hypotheses delimiting these taxa (Kinzey, 1982; Rylands et al., 1988; Gregorin, 2006; Povill et al., 2023), we found no support for any (Figure 4; Supplementary Materials 4, 5).

Based on the study of mtDNA CytB by Harris et al. (2005), Fortes and Bicca-Marques (2008) suggested that *A. g. clamitans* could be, in fact, two species. These authors used samples from SC, RJ, and SP, and they also found SP haplotypes in both clades, which supports our results. Probably due to a limited sampling (N = 15), they found only two lineages. The study by de Mello-Martins et al. (2011), based on coalescent analysis using mtDNA CytB (N = 38) from three Brazilian states (RJ, SP, and SC), found two distinct haplogroups corresponding to the RJ and SC populations, with divergent haplogroups occurring in sympatry in the same population in São Paulo. Surprisingly, these authors also interpreted this result as *A. guariba clamitans* possibly constituting two subspecies or two potential species, as had Harris et al. (2005). Mello-Martins et al. (2011), however, mentioned that analyses of genetic variation at multiple autosomal genetic markers were required to test the said taxonomic hypothesis. Our STR analysis clearly showed that there is no reproductive isolation between SP populations/individuals and no substructure within the state (Figure 2).

More recently, Povill et al. (2023) analyzed the mtDNA CytB gene of 14 captive and 108 free-living individuals, ten karyotypes, and ecological niche models and suggested the validity of two taxa (which they named using the currently recognized subspecies) but argue that the limit is further south, in the state of São Paulo, although they do not specify exactly where. Their proposal of a northern (individuals in their “northern clade,” A + B clades) and a southern clade (those with C clade haplotypes) is not supported by our or previous data. First, there is no consistent geographical, morphological, or, as shown here, genetic (at the nuclear level) attribute, apart from the mtDNA clades, to separate individuals with haplotypes from A + B versus those with C haplotypes. Second, because A, B, and C haplotypes are present in the central region (SP), even in the same populations, using their criteria (that individuals with mtDNA from clade (A + B) are from one species and those with mtDNA from clade C are from another), we would have several populations with individuals from the two species that otherwise are not distinguishable. In addition, although of minor importance to our above arguments, an mtDNA “northern clade” (clades A + B) is not supported by our more informative phylogeny obtained from our combined CR + CytB dataset (Supplementary Material 5). In summary, as they correctly stated, although mtDNA is useful to help understand some phylogenetic and phylogeographic patterns, it is a single, uniparental inherited loci. Species delimitations should be based, alongside other important information, on more (independent) loci, as done here, and ideally on a genomic dataset.

Gregorin’s (2006) proposal that *A. g. guariba* is a monotypic species (*A. fusca*, see introduction) restricted to the northernmost region of the brown howler range (northern RJ, ES, and southern BA) is also not supported by our results (Figure 4). Although our samples from this region presented the same nuclear genetic component (purple), samples from other areas that would be classified as *A. g. clamitans* (*A. clamitans* by his criteria) also presented this genetic component (purple). Furthermore, samples from his *A. fusca* region have diverse mtDNA haplotypes that interspersed in the tree among haplotypes found in putatively *A. g. clamitans*, including some haplotypes that are identical in both of his species. These findings also negate the suggestions that *A. g. guariba* has a southerly limit north of the Rio Doce (Kinzey, 1982) or occurs only north of the Rio Jequitinhonha basin (Rylands et al., 1988; Neves et al., 2018).

In conclusion, we did not corroborate previous taxonomic proposals to separate *A. guariba* into two species or subspecies, and we consider that the Atlantic Forest brown howler monkeys comprise a single species, *A. guariba*, without infraspecific taxonomic entities. However, we show that this single taxonomic unit supported by our results is genetically structured in both nuclear and mtDNA markers but in a complex way. The southern group (from RS + SC) is homogeneous both in the STR and mtDNA markers. The set of populations from the central area, especially from SP, is relatively homogeneous for the STR, with its own genetic component (Figure 2); however, it presents all three mtDNA clades (Figures 4, 5A; Supplementary Material 5). The northern set of populations, from ES and MG, is more heterogeneous for both the STR and mtDNA data. This geographic structure is corroborated by other evidence. For example, multiple intraspecific chromosomal rearrangements have been found in A. guariba, possibly distributed in a clinal gradient (Oliveira et al. 2000; Steinberg et al., 2017). Individuals from the southern states of SC and PR have a different diploid numbers, sex chromosome systems and different chromosomal rearrangements compared to individuals from central (2n = 45♂/46♀, X_1_X_2_X_3_Y_1_Y_2_ or X_1_X_2_Y /X_1_X_1_X_2_X_2_X_3_X_3_ or X_1_X_1_X_2_X_2_ versus 2n = 49 ♂/48 or 50 ♀, XY or X_1_X_2_X_3_Y_1_Y_2_ or X_1_X_2_Y/ XX or X_1_X_1_X_2_X_2_X_3_X_3_ or X_1_X_1_X_2_X_2_) or northern populations (2n = 49 or 52 ♂/50 ♀, XY or X_1_X_2_Y/ X_1_X_1_X_2_X_2_) (Koiffmann 1977; Oliveira et al. 2000; 2002; Steinberg et al., 2017). However, this is not indicative of taxonomic differences either, because howler monkeys show a high degree of karyological variation within species (Koiffmann, 1977; Gifalli-Iughetti, 2008), as observed in many other mammalian taxa (e.g., some rodents of the genus *Ctenomys*; Lopes et al., 2013 and references therein). In addition, populations located between the core areas of the three groups, such as RJ between the northern and central groups and ARG between the central and southern groups, seem genetically intermediate or admixed for the STRs.

### 4.3 Phylogeographic patterns and demographic processes

The interpretation of *A. guariba* dynamics in the Atlantic Forest could be compared with several phylogeographic studies suggesting two main recurrent breaks in its range, (i) one in São Paulo, near the valleys of the rivers Paraíba do Sul, Tietê, and Ribeira do Iguape (reported for snakes, toads, birds, frogs, and bats; Grazziotin et al., 2006; Cabanne et al., 2007; 2008; 2011; Carnaval et al., 2009; Martins et al., 2009; Batalha-Filho et al., 2010; Thomé et al., 2010; d’Horta et al., 2011); (ii) another in Minas Gerais, near the valleys of the rivers Jequitinhonha and Doce (bird and frogs; Cabanne et al., 2007; 2008; Carnaval et al., 2009; d’Horta et al., 2011). For the primates, these would correspond to the Paulista and Bahia centers of endemism identified by Kinzey (1982) and Rylands et al. (1996).

One well-known hypothesis about the origin of diversity in the Neotropical region is the refuge theory. According to this theory, proposed for the Amazonian basin and expanded to the Atlantic Forest, during the glacial ages, the rainforests were reduced to refuges isolated by open areas, and organisms isolated in these refuges could have diverged and originated new lineages. Then, in the next interglacial period, the forest expanded, bringing the new clades into contact (Haffer, 1969; Carnaval and Moritz, 2008; Carnaval et al., 2009). Following this reasoning, one possible explanation for the existence of three very divergent mtDNA in the species is that these clades could represent a differentiation of the populations due to vicariant events during the forest fragmentation followed by expansions in favorable periods that induced secondary contacts (represented here by the SP populations). Indeed, our BSP demographic results (Figure 5B) support this hypothesis, as the three mtDNA clades are mostly geographically structured and went through a process of population expansion (see below). However, mitochondrial markers are maternally inherited; therefore, they will present a bias if the dispersion is not equal for both sexes. In most *Alouatta* species, demographic records show that, in continuous forests, both female and male species disperse (Kinzey, 1997); however, habitat fragmentation has been shown to modify the dispersal pattern in *A. caraya* from bisexual to male-biased (Oklander et al., 2010), increasing female philopatry that could cause an increase in phylogeographic structuring of mtDNA and disagreement between it and nuclear markers. The Atlantic Forest has been fragmented historically with open areas during the Pleistocene (Behling, 2002), and those patches of forest were widely isolated (Behling and Negrelle, 2001). This fragmentation could have led to the isolation of matrilines that could have been important in shaping the mitochondrial clades observed here.

The Carnaval and Moritz (2008) model of Pleistocene refugia predicts a large stable area in the north of the Atlantic Forest (latitude 28°/27°S to at least 20°S) and very small forest fragments in the south during the Last Glacial Maximum (LGM, 18 to 48 kya), with fragmentation causing isolation between areas that could be detected by mitochondrial DNA markers. It also predicts significant signals of demographic expansion in the southern part of the Atlantic Forest, but an absence of demographic expansion signals in the northern and central portions caused by the permanence of the humid forest. Our results on STRs agree with the latitudinal gradient hypothesis, which states that higher diversity levels should be found in lower latitudes (Miller et al., 2010; d’Horta et al., 2011). However, for mitochondrial markers, greater variability is observed in São Paulo, decreasing toward the north and south in general terms.

Different from what was expected by the Carnaval and Moritz model depicted above (demographic stability in the north and reduction followed by expansion in the south), if we analyze the three mtDNA clades separately, the southern (C) and the northern (B and C) clades have a very similar BSP demographic trajectory of an expansion starting between 40 and 20 kya during the LGM (Behling, 2002; Behling et al., 2004), followed by a recent reduction approximately between 4 and 2 kya. This result suggests the demographic consequences of the climatic events in the last 50 kya were similar along the distribution of the species. A possible explanation for the estimated population expansion in a likely unfavorable period is the humidity variation during the Pleistocene that formed ecoregions favorable for this species. Palynological studies have shown that grassland predominated in southern and southeastern Brazilian regions during the Late Pleistocene, mainly during the LGM, when tropical trees were almost absent (Behling, 2002; Behling et al., 2004). However, the pollen data suggest that small populations of *Araucaria* and the Atlantic rainforest trees may have been present on the wetter coastal slopes and deep and protected river valleys that may have served as a refuge (Behling et al., 2004). In addition, periods of increased humidity favored forests of conifers, which appear to have expanded during the glaciations when the climate was cool and moist enough for their development, forming mosaics with grassland (Ledru et al., 1996; Ledru et al., 2007; Pessenda et al., 2009). Thus, the generalist diet of *A. guariba*, which is composed of numerous plant species, including conifers such as *Araucaria angustifolia* (Miranda and Passos, 2004; Martins, 2008), could have favored its survival in the expanded conifer forest and their associated vegetation. The existence of the central population (SP) with haplotypes of the three clades may be explained as a secondary contact after the expansions of the northern and southern populations from their putative refugia.

We also found evidence that *A. guariba* suffered a strong and recent population reduction beginning between 4 and 2 kya. This period was marked by humidity and temperature oscillations. For example, Garcia et al. (2004) and Behling et al. (2005) showed that in SP and RS, between ∼10 and ∼4 kya, the climate was much drier than today. These oscillations in humidity and temperature could have affected the demography of *A. guariba*. This long-term population decline (Figure 5B) may have been accelerated recently by anthropogenic changes and habitat fragmentation.

Finally, the six major river basins in the Atlantic Forest are frequently suggested as barriers to gene flow and sources of population divergence (Cabanne et al., 2008). However, our data do not support their influence as important barriers to gene flow in *A. guariba*, as the geographic breaks between our three major nuclear genetic components are not consistent with the course of any of these rivers (Figure 2B).

### 4.4 Conservation implications

Brown howler monkeys are important seed dispersers for numerous plant species as they feed on a wide diversity of fruits, thus having a crucial role in the maintenance and regeneration of forests throughout their range (Chaves et al., 2018). Due to their susceptibility to yellow fever, brown howlers also play a key role as public health sentinels, providing early warning of the virus circulation and allowing for pre-emptive vaccination of surrounding human populations, especially because the species’ range largely overlaps with many of the most inhabited areas of Brazil. Howlers are also part of the cultural heritage of both native people and settlers inhabiting Brazil and Argentina (Urbani and Cormier, 2015). The conservation of this species, therefore, contributes to the regeneration of the flora of the Atlantic Forest, to preserving their cultural heritage, and to promoting public health and awareness toward biodiversity and habitat conservation.

Although we argue that our results advise against any taxonomic subdivision for the species, the pattern of differentiation depicted in the structure analysis suggests these two or three genetic groups maintain enough differentiation to deserve appropriate conservation strategies, including population management (Oklander et al., 2024). These dissimilar genetic structures, found in nuclear markers and mtDNA, may be indicative of processes of differentiation and reestablishment of gene flow at different times but do not indicate a clear taxonomic differentiation. These differences can, however, be important from a conservation point of view, and therefore, they deserve to be treated as different conservation units. Conservation units could be (i) evolutionarily significant units (ESUs—Moritz, 1994) that are based on reciprocal monophyly at mitochondrial markers or (ii) management units (MUs) identifiable by significant differences in allele frequency distributions and significant divergences in mitochondrial or nuclear loci. Considering these criteria, we propose three MUs based on our results:

• MU1—RJ, ES, MG, and BA: MG, ES, and RJ share a nuclear component purple (Figure 2). MG and BA are grouped in two mitochondrial clades (A and B), and RJ is grouped in clade B in our sampling, but in both clades (A and B) in the CytB tree when RJ sequences from Povill et al. (2023) are included. BA is grouped in mitochondrial clade A with MG and ES, but we do not have nuclear data for this state. A management unit (MU) grouping of MG, ES, and RJ is currently adopted for population management (Oklander et al., 2024), and we are suggesting the inclusion of BA. We recommend, however, that the management of the populations in these states be carried out with caution because sampling is limited regarding geographic representativeness for MG (especially by the gap in the southeast region), the number of individuals for RJ (N = 4), and the paucity of molecular markers analyzed for BA (one site, one individual, mtDNA data only). We thus suggest, as a precaution, the management of the populations of these areas independently until more data are available.
• MU2—SP: The STR analysis indicates it has its own component (yellow, Figure 2), sharing some variability with RJ and ARG, and all three mitochondrial clades (A, B, and C, Figures 4, 5; Supplementary Material 5) were found there, thus presenting variability shared with populations along the species distribution. So, in general terms, it could receive individuals from any location that would not be significantly different from its own. On the other hand, being so variable, SP individuals could carry exotic variability when being moved out of the state. Hence, we suggest that it may be considered an MU in itself. Although this state has the third largest number of sites (N = 11) and individuals (N = 43) sampled, almost half of the samples are from a single locality (Serra da Cantareira, N = 19), many locations (N = 5) are represented by a single sample, and the central and western ranges of the species in the state were not sampled. Expanding this sampling, therefore, can help increase the resolution of the genetic diversity present in SP and provide further guidance for management measures.
• MU3—ARG, RS, SC, and PR: In the STR analysis, they are grouped as component green (Figure 2) with the exception of PR, for which we only have mitochondrial data. They are also grouped in the mitochondrial clade C. This MU is the same as the one currently adopted to guide population management (Oklander et al., 2024) but includes PR. There are sampling gaps in northern RS and western SC, even though they are the states with most sites (RS = 20; SC = 19) and individuals (RS = 57; SC = 53) sampled for this study. Furthermore, even though we only have five samples from one site in Argentina, this represents more than 10% of the remaining individuals in the country (estimated between 30 and 50, Agostini et al., 2019). Probably the most relevant sampling gap for this MU is, therefore, in east and central PR, for which we only analyzed mtDNA from a single locality in the extreme west of the state. Thus, the translocation of individuals from PR to other states should be treated with caution until information on other populations, mainly from central and eastern PR, is available. However, given the urgency of implementing management actions to recover populations in Argentina and avoid the near extinction of the species there (Oklander et al., 2024), the inclusion of animals from western PR—especially if there are no others available in RS and/or SC—in these translocations can be considered an acceptable exception.

In terms of the global conservation status of *A. guariba* as a species, the present results do not support changes in its current categorization as Vulnerable (Jerusalinsky et al., 2021), nor in the existing recommendation to uplist the species to Endangered. This is because the absence of infraspecific taxonomic divisions in *A. guariba*, as proposed here, does not change the known total size of its population and decline trend, extent of occurrence, and area of occupancy, nor the various threats known to impact the species along its distribution. However, the status of the species at the regional level (Brazilian states and nationally in Argentina) should be reevaluated as a single taxonomic unit. This is particularly important to MG and ES because both the previously recognized subspecies were assumed to occur in these states, influencing the available regional assessments. On the other hand, although regular updates on the regional status are always recommended, for most assessments at the state or national level, the category assigned to the previously recognized subspecies should probably be maintained when evaluating these populations as a single species because only one of these subspecies was considered to occur in Argentina and most Brazilian states (BA, RJ, SP, PR, SC, and RS). Official assessments from three states—Rio Grande do Sul (Decreto Estadual No. 51.797, 2014), Santa Catarina (Resolução Consema No. 002, 2011), and Minas Gerais (Deliberação Normativa COPAM No. 147, 2010)—assigned *A. guariba clamitans* the same current category as the species: Vulnerable. The risk was considered higher in the other regional assessments: *A. guariba clamitans* was evaluated as Critically Endangered in Argentina (Agostini et al., 2019; MAyDS, 2021) and Endangered in the states of São Paulo (Decreto Estadual No 63,853, 2018) and Espírito Santo (Decreto Estadual No. 5.237/2022); and *A. guariba guariba* was classified as Critically Endangered in Brazil (Neves et al., 2018) and in the states of Minas Gerais (Deliberação Normativa COPAM no 147, 2010) and Bahia (Portaria no 37, 2017).

Now, considering *A. guariba* as a monotypic species with three management units, we suggest equal and synchronized conservation strategies for the three MUs described here, following the national action plans for the species in Brazil and Argentina. The fact that the northern part of the distribution does not consist of a different species does not diminish the importance of prioritizing the conservation of these populations, which are extremely scarce and vanishing in southern BA, northern ES, and northeastern MG (Neves et al., 2019). Moreover, the conservation of each population of *A. guariba* is relevant because they are threatened by a variety of menaces and declining all along their range, justifying its inclusion among the world’s most endangered primates (Oklander et al., 2022). The impacts of synergistic threats, which led to species extirpation or severe decline in many areas, highlight the importance of developing population management strategies properly based on the conservation units identified here (Oklander et al., 2024). Finally, the implementation of other conservation strategies, such as the creation of protected areas and the establishment of forest corridors, as stated in the national action plans for the species, must also incorporate the present results to effectively preserve part of the biodiversity represented by the intraspecific genetic structure and variability of *A. guariba*.

## Data Availability

The datasets presented in this study can be found in online repositories. The names of the repository/repositories and accession number(s) can be found in the article/Supplementary Material.

## References

[B1] Agostini I. Holzmann I. Oklander L. I. Peker S. Pavé R. Kowalewski M. (2019). “ *Alouatta guariba* in Categorización 2019 de los mamíferos de Argentina según su riesgo de extinción. *Red List of Mammals in Argentina* ,” in SAyDS–SAREM (Buenos Aires, Argentina). 10.31687/SaremLR.19.117

[B2] Almeida M. A. B. Santos E. Cruz Cardoso J. Fonseca D. F. Noll C. A. Silveira V. R. (2012). Yellow fever outbreak affecting *Alouatta* populations in southern Brazil (Rio Grande do Sul State), 2008–2009. Am. J. Primatol. 74 (1), 68–76. 10.1002/ajp.21010 22020690

[B3] Ascunce M. S. Cortez-Ortiz L. Mudry M. D. (2003). The mitochondrial control region of the black howler monkey, *Alouatta caraya* (Primates, Platyrrhini), and the development of new primers. Mol. Ecol. Notes 3, 372–375. 10.1046/j.1471-8286.2003.00454.x

[B4] Batalha-Filho H. Waldschmidt A. M. Campos L. A. O. Tavares M. G. Fernandes-Salomão T. M. (2010). Phylogeography and historical demography of the Neotropical stingless bee *Melipona quadrifasciata* (Hymenoptera, Apidae): incongruence between morphology and mitochondrial DNA. Apidologie 41, 534–547. 10.1051/apido/2010001

[B5] Behling H. (2002). South and southeast Brazilian grasslands during Late Quaternary times: a synthesis. Palaeogeogr. Palaeoclimatol. Palaeoecol. 177, 19–27. 10.1016/s0031-0182(01)00349-2

[B6] Behling H. Negrelle R. R. B. (2001). Tropical rain forest and climate dynamics of the Atlantic lowland, southern Brazil, during the late Quaternary. Quat. Res. 56, 383–389. 10.1006/qres.2001.2264

[B7] Behling H. Pillar V. P. Bauermann S. G. (2005). Late Quaternary grassland (Campos), gallery forest, fire and climate dynamics, studied by pollen, charcoal and multivariate analysis of the São Francisco de Assis core in western Rio Grande do Sul (southern Brazil). Rev. Palaeobot. Palynol. 133, 235–248. 10.1016/j.revpalbo.2004.10.004

[B8] Behling H. Pillar V. P. Orlóci L. Bauermann S. G. (2004). Late Quaternary *Araucaria* Forest, grassland (Campos), fire and climate dynamics, studied by high-resolution pollen, charcoal and multivariate analysis of the Cambará do Sul core in southern Brazil. Palaeogeogr. Palaeoclimatol. Palaeoecol. 203, 277–297. 10.1016/s0031-0182(03)00687-4

[B9] Bicca-Marques J. C. Alves S. L. Ingberman B. Buss G. Fries B. G. Alonso A. C. (2018). “ Alouatta guariba clamitans ,” in Livro Vermelho da Fauna Brasileira Ameaçada de Extinção: Volume II – Mamíferos. Editors Machado A. B. M. Drummond G. M. Paglia A. (Brasília: Instituto Chico Mendes de Conservação da Biodiversidade ICMBio).

[B10] Bouckaert R. Vaughan T. G. Barido-Sottani J. Duchêne S. Fourment M. Gavryushkina A. (2019). BEAST 2.5: an advanced software platform for Bayesian evolutionary analysis. PLoS Comput., Biol. 15 (4), e1006650. 10.1371/journal.pcbi.1006650 30958812 PMC6472827

[B11] Buss G. Bicca-Marques J. C. Alves S. L. Ingberman B. Fries B. G. Alonso A. C. (2021). Alouatta guariba *ssp.* clamitans *(amended version of the 2020 assessment)* . Gland, Switzerland: The IUCN Red List of Threatened Species. 2021: e.T39918A190419216.

[B12] Cabanne G. S. D’Horta F. M. Meyer D. Silva J. M. C. Miyaki C. Y. (2011). Evolution of *Dendrocolaptes platyrostris* (aves: furnariidae) between the south American open vegetation corridor and the atlantic forest. Biol. J. Linn. Soc. 103, 801–820. 10.1111/j.1095-8312.2011.01678.x

[B13] Cabanne G. S. d’Horta F. M. Sari E. H. R. Santos F. R. Miyaki C. Y. (2008). Nuclear and mitochondrial phylogeography of the Atlantic Forest endemic *Xiphorhynchus fuscus* (Aves: Dendrocolaptidae): biogeography and systematic implications. Mol. Phylogenet. Evol. 49, 760–773. 10.1016/j.ympev.2008.09.013 18849002

[B14] Cabanne G. S. Santos F. R. Miyaki C. Y. (2007). Phylogeography *of Xiphorhynchus fuscus* (Passeriformes, Dendrocolaptidae): vicariance and recent demographic expansion in southern Atlantic Forest. Biol. J. Linn. Soc. 91 (1), 73–84. 10.1111/j.1095-8312.2007.00775.x

[B15] Cabrera A. (1940). Los nombres científicos de algunos monos americanos. Cienc. Mex. 1, 402–405.

[B16] Cabrera A. (1957). Catálogo de los mamíferos de América del Sur. Rev. Mus. Argent. Cienc. Nat. Bernardino Rivadavia, Zool. Buenos Aires 4, 1–307.

[B17] Câmara I. de G. (1991). Plano de Ação para a Mata Atlântica. São Paulo: SOS Mata Atlântica, Scritta Oficina Editorial Ltda.

[B18] Carnaval A. C. Hickerson M. J. Haddad C. F. Rodrigues M. T. Moritz C. (2009). Stability predicts genetic diversity in the Brazilian Atlantic Forest hotspot. Science 323 (5915), 785–789. 10.1126/science.1166955 19197066

[B19] Carnaval A. C. Moritz C. (2008). Historical climate modelling predicts patterns of current biodiversity in the Brazilian Atlantic forest. J. Biogeog. 35, 1187–1201. 10.1111/j.1365-2699.2007.01870.x

[B20] Caye K. Deist T. M. Martins H. Michel O. François O. (2016). TESS3: fast inference of spatial population structure and genome scans for selection. Mol. Ecol. Res. 16 (2), 540–548. 10.1111/1755-0998.12471 26417651

[B21] Chaves Ó. M. Bicca-Marques J. C. Chapman C. A. (2018). Quantity and quality of seed dispersal by a large arboreal frugivore in small and large Atlantic Forest fragments. PLoS One 13 (3), e0193660. 10.1371/journal.pone.0193660 29561869 PMC5862440

[B22] Chaves Ó. M. Júnior J. S. Buss G. Hirano Z. M. Jardim M. M. A. Amaral E. L. S. (2022). Wildlife is imperiled in peri-urban landscapes: threats to arboreal mammals. Sci. Total Environ. 821, 152883. 10.1016/j.scitotenv.2021.152883 35038525

[B23] Corander J. Marttinen P. Sirén J. Tang J. (2008). Enhanced Bayesian modelling in BAPS software for learning genetic structures of populations. BMC Bioinf 9, 539. 10.1186/1471-2105-9-539 PMC2629778 19087322

[B24] d’Horta F. M. Cabanne G. S. Meyer D. Miyaki C. Y. (2011). The genetic effects of Late Quaternary climatic changes over a tropical latitudinal gradient: diversification of an Atlantic Forest passerine. Mol. Ecol. 20, 1923–1935. 10.1111/j.1365-294X.2011.05063.x 21410807

[B25] Di Fiore A. Fleischer R. C. (2004). Microsatellite markers for wooly monkeys (*Lagothrix lagotricha*) and their amplification in other New World primates (Primates: Platyrrhini). Mol. Ecol. Notes 4, 246–249. 10.1111/j.1471-8286.2004.00631.x

[B26] Drummond A. J. Rambaut A. Shapiro B. Pybus O. G. (2005). Bayesian coalescent inference of past population dynamics from molecular sequences. Mol. Biol. Evol. 22 (5), 1185–1192. 10.1093/molbev/msi103 15703244

[B27] Endicott P. Ho S. Y. (2008). A Bayesian evaluation of human mitochondrial substitution rates. Am. J. Hum. Genet. 82 (4), 895–902. 10.1016/j.ajhg.2008.01.019 18371929 PMC2427281

[B28] Evanno G. Regnau S. Goudet J. (2005). Detecting the number of clusters of individuals using the software Structure: a simulation study. Mol. Ecol. 14, 2611–2610. 15969739 10.1111/j.1365-294X.2005.02553.x

[B29] Excoffier L. Lischer H. E. L. (2010). Arlequin suite ver 3.5: a new series of programs to perform population genetics analyses under Linux and Windows. Mol. Ecol. Res. 10, 564–567. 10.1111/j.1755-0998.2010.02847.x 21565059

[B30] Farris J. S. Kallersjo M. Kluge A. G. Bult C. (1995). Constructing a significance test for incongruence. Syst. Biol. 44 (4), 570–572. 10.2307/2413663

[B31] Fortes V. B. Bicca-Marques J. C. (2008). Abnormal pelage color in an isolated population of *Alouatta guariba clamitans* Cabrera, 1940 in south Brazil. Int. J. Primatol. 29, 717–722. 10.1007/s10764-008-9264-7

[B32] Francis R. M. (2016). pophelper: an R package and web app to analyse and visualize population structure. Mol. Ecol. Res. 17, 27–32. 10.1111/1755-0998.12509 26850166

[B33] Fu Y. X. (1997). Statistical tests of neutrality of mutations against population growth, hitchhiking and background selection. Genetics 147, 915–925. 10.1093/genetics/147.2.915 9335623 PMC1208208

[B34] Garcia M. J. Oliveira P. E. Siqueira E. Fernandes R. S. (2004). A Holocene vegetational and climatic record from the Atlantic rainforest belt of coastal State of São Paulo, SE Brazil. Rev. Palaeobot. Palynol. 131, 181–199. 10.1016/j.revpalbo.2004.03.007

[B35] Geoffroy Saint-Hilaire É. (1806). Memoire sur les singes a main imparfaite ou les ateles. Ann. Mus. Hist. Nat. Paris 7, 260–273.

[B36] Gifalli-Iughetti C. (2008). *Evolução Cromossômica: Estudo da Variabilidade Cariotípica em Platyrrhini e das Homeologias e Sintenias com Cromossomos Humanos* . Tesis Doctoral. São Paulo, Brazil: Universidade de São Paulo.

[B37] Gonçalves E. C. Silva A. Barbosa M. S. R. Schneider M. P. C. (2004). Isolation and characterization of microsatellite loci in Amazonian red-handed howlers *Alouatta belzebul* (Primates, Plathyrrini). Mol. Ecol. Notes 4, 406–408. 10.1111/j.1471-8286.2004.00667.x

[B38] Goudet J. (2003). Fstat (ver. 2.9.4). A program to estimate and test population genetics parameters. Available at: http://www.unil.ch/izea/softwares/fstat.html.

[B39] Grazziotin F. G. Monzel M. Echeverrigaray S. Bonatto S. L. (2006). Phylogeography of the *Bothrops jararaca* complex (Serpentes: Viperidae): past fragmentation and island colonization in the Brazilian Atlantic Forest. Mol. Ecol. 15, 3969–3982. 10.1111/j.1365-294X.2006.03057.x 17054497

[B40] Gregorin R. (2006). Taxonomia e variação geográfica das espécies do gênero *Alouatta* Lacépède (Primates, Atelidae) no Brasil. Revta. Bras. Zool. 23, 64–144. 10.1590/s0101-81752006000100005

[B41] Groves C. P. (2001). Primate taxonomy. Washington, DC: Smithsonian Institution Press.

[B42] Groves C. P. (2005). “Order Primates,” in Mammal species of the world: a taxonomic and geographic reference. 3rd Edn, Editors Wilson D. E. Reeder D. M. (Baltimore, MD: Johns Hopkins University Press), 1, 111–184.

[B43] Haffer J. (1969). Speciation in Amazonian forest birds. Science 165, 131–137. 10.1126/science.165.3889.131 17834730

[B44] Harris E. E. Iughtti C. G. Braga Z. H. Koiffmann C. P. (2005). Cytochrome b sequences show subdivision between populations of the brown howler monkey (*Alouatta guariba*) from Rio de Janeiro and Santa Catarina, Brazil. Neotrop. Prim. 13, 16–21.

[B45] Hershkovitz P. (1963). *Primates: comparative anatomy and taxonomy, V. Cebidae, Part B.* A monograph by W. C. Osman Hill. Edinburgh University Press. 1962. xxix 537pp., 34 pls. 94 figs., 3 maps. A critical review with a summary on the volumes on New World primates. American Journal of Biological Anthropology 21 (3), 391–398. 10.1002/ajpa.1330210318

[B46] Hill W. C. O. (1962). Primates: comparative anatomy and taxonomy V. Cebidae Part B. Edinburgh: Edinburgh University Press.

[B47] Hirano Z. M. B. (2004). Secreção epidérmica de *Alouatta guariba clamitans* (Primates: Atelidae). Tese de Doutorado. Faculdade de Filosofia, Ciências e Letras de Ribeirão Preto (Ribeirão Preto, Brazil: Universidade de São Paulo).

[B48] Hirano Z. M. B. Tramonte R. Silva A. R. Rodrigues R. B. Santos W. F. (2003). Morphology of epidermal glands responsible for release of colored secretions in *Alouatta guariba clamitans* . Lab. Primates Newsl. 42 (2), 4–6.

[B49] Humboldt A. von. 1811 (1812). “Tableau synoptique des singes de l'Amerique,” in A. J. Recueil d'observations de zoologie et d'anatomie comparee, faites dans l'ocean Atlantique, dans l'interieur du nouveau continent et dans la mer du sud pendant les annees 1799[–]1803, Premier volume, Deuxieme partie, Observations de zoologie et d'anatomie comparee (Paris: Schoell and Dufour). Editors von Humboldt, A. Bonpland A., 353–363. [For date of publication, see Sherborn (1899].

[B50] ICMBio (2018). Portaria no 702, de 7 de agosto de 2018 - Aprova o Plano de Ação Nacional para a Conservação dos Primatas da Mata Atlântica e da Preguiça-de-Coleira. Instituto Chico Mendes de Conservação da Biodiversidade. Diário Oficial da União 153 - Seção 1, 57.

[B51] IPS & APS (2014). Code of best Practices for field Primatology. International Primatological Society and American Society of Primatologists, 17. Available at: https://internationalprimatologicalsociety.org/wp-content/uploads/2021/10/Code-of_Best_Practices-Oct-2014.pdf.

[B52] Irwin D. M. Kocher T. D. Wilson A. C. (1991). Evolution of the cytochrome b gene of mammals. J. Mol. Evol. 32, 128–144. 10.1007/BF02515385 1901092

[B53] Jerusalinsky L. (2001). *Diversidade em Sequências Mitocondriais do Bugio-ruivo* (Alouatta guariba): *Implicações para a História Evolutiva e a Conservação da Espécie* . Masters Dissertation. Porto Alegre, Brazil: Universidade Federal do Rio Grande do Sul. 80.

[B54] Jerusalinsky L. Bicca-Marques J. C. Neves L. G. Alves S. L. Ingberman B. Buss G. (2021). Alouatta guariba *ssp.* guariba *(amended version of the 2021 assessment)* . *The IUCN Red List of Threatened Species 2021:* e.T39916A190417874. United Kingdom: IUCN Red List. 2021.

[B55] Jombart T. Ahmed I. (2011). Adegenet 1.3-1: new tools for the analysis of genome-wide SNP data. Bioinformatic 27 (21), 3070–3071. 10.1093/bioinformatics/btr521 PMC3198581 21926124

[B56] Kinzey W. G. (1982). “Distribution of primates and forest refuges,” in Biological diversification in the tropics. Editor Prance G. T. (New York: Columbia University Press), 455–482.

[B57] Kinzey W. G. (1997). New world primates: Ecology, evolution, and behavior. New York: Aldine de Gruyter.

[B58] Koiffmann C. P. (1977). Variabilidade cromossómica na família cebidae (Platyrrhini, primata). Sao Paulo: Instituto de Biociencias, Universidade de São Paulo. Ph.D. thesis.

[B59] Kumar S. Stecher G. Tamura K. (2016). MEGA7: molecular evolutionary genetics analysis version 70 for bigger datasets. Mol. Biol. Evol. 33 (7), 1870–1874. 10.1093/molbev/msw054 27004904 PMC8210823

[B60] Ledru M. P. Braga P. I. S. Soubiès F. Fournier M. Martin L. Suguio K. (1996). The last 50,000 years in the Neotropics (Southern Brazil): evolution of vegetation and climate. Palaeogeogr. Palaeoclimatol. Palaeoecol. 123 (1–4), 239–257. 10.1016/0031-0182(96)00105-8

[B61] Ledru M. P. Salatino M. L. F. Ceccantini G. Salatino A. Pinheiro F. Pintaud J. C. (2007). Regional assessment of the impact of climatic change on the distribution of a tropical conifer in the lowlands of South America. Divers. Distrib. 13 (6), 761–771. 10.1111/j.1472-4642.2007.00389.x

[B62] Lister A. M. (2004). The impact of Quaternary ice ages on mammalian evolution. Phil. Trans. Roy. Soc. B 359, 221–241. 10.1098/rstb.2003.1436 15101579 PMC1693321

[B63] Lopes C. M. Ximenes S. S. F. Gava A. De Freitas T. R. O. (2013). The role of chromosomal rearrangements and geographical barriers in the divergence of lineages in a South American subterranean rodent (Rodentia: Ctenomyidae: *Ctenomys minutus*). Heredity 111 (4), 293–305. 10.1038/hdy.2013.49 23759727 PMC3807268

[B64] Machado S. (2011). *Filogeografia do Bugio-Ruivo,* Alouatta guarba *(Primates, Atelidae)* . Masters dissertation. Porto Alegre, Brazil: Pontifícia Universidade Católica do Rio Grande do Sul. 37.

[B65] Martins F. M. Templeton A. R. Pavan A. C. O. Kohlbach B. C. Morgante J. S. (2009). Phylogeography of the common vampire bat (*Desmodus rotundus*): marked population structure, Neotropical Pleistocene vicariance and incongruence between nuclear and mtDNA markers. BMC Evol. Biol. 9, 294. 10.1186/1471-2148-9-294 20021693 PMC2801518

[B66] Martins M. M. (2008). Fruit diet of *Alouatta guariba* and *Brachyteles arachnoides* in southeastern Brazil: comparison of fruit type, color, and seed size. Primates 49, 1–8. 10.1007/s10329-007-0050-5 17578654

[B67] MAyDS (2021). Resolución 430/2021 - Aprueba el Plan Nacional de Conservación de Primates de la Argentina. Minist. Ambiente Desarro. Sosten. Available at: https://www.boletinoficial.gob.ar/detalleAviso/primera/255189/20211227.

[B68] Mello-Martins F. Gifalli-Iughetti C. Koiffmann C. P. Harris E. E. (2011). Coalescent analysis of mtDNA indicates Pleistocene divergence among three species of howler monkey (*Alouatta* spp.) and population subdivision within the Atlantic Coastal Forest species. A. Guariba. Primates 52 (1), 77–87. 10.1007/s10329-010-0226-2 21107992

[B69] Miller M. J. Bermingham E. Klicka J. Escalante P. Winker K. (2010). Neotropical birds show a humped distribution of within-population genetic diversity along a latitudinal transect. Ecol. Lett. 13, 576–586. 10.1111/j.1461-0248.2010.01454.x 20529101

[B70] Miranda J. Passos F. C. (2004). Feeding habits of *Alouatta guariba* (Humboldt) (primates, atelidae) in *Araucaria* pine forest, Paraná, Brazil. Rev. Bras. Zool. 21, 821–826. 10.1590/s0101-81752004000400016

[B71] Mittermeier R. A. Rylands A. B. Wilson D. E. (2013). Handbook of the mammals of the world. 3, *Primates*. (Barcelona: Lynx Edicions).

[B72] Moreno E. S. Agostini I. Holzmann I. Di Bitetti M. S. Oklander L. I. Kowalewski M. M. (2015). Yellow fever impact on brown howler monkeys (*Alouatta guariba clamitans*) in Argentina: a metamodelling approach based on population viability analysis and epidemiological dynamics. Mem. Inst. Oswaldo Cruz 110 (7), 865–876. 10.1590/0074-02760150075 26517499 PMC4660615

[B73] Moritz C. (1994). Applications of mitochondrial DNA analysis in conservation: a critical review. Mol. Ecol. 3, 401–411. 10.1111/j.1365-294x.1994.tb00080.x

[B74] Mourthé I. Trindade R. A. Aguiar L. M. Trigo T. C. Bicca-Marques J. C. Bonatto S. L. (2019). Hybridization between Neotropical primates with contrasting sexual dichromatism. Int. J. Primatol. 40, 99–113. 10.1007/s10764-017-0011-9

[B75] Neves L. G. Jerusalinsky L. Melo F. R. (2018). Livro Vermelho da Fauna Brasileira Ameaçada de Extinção: Volume II – Mamíferos, Org. Instituto Chico Mendes de Conservação da Biodiversidade – ICMBio. (Brasilia: Instituto Chico Mendes de Conservacao da Biodiversidade ICMBio), 162–166.

[B76] Neves L. G. Jerusalinsky L. Talebi M. Mittermeier R. A. Cortés-Ortiz L. Melo F. R. (2021). Alouatta guariba *ssp.* guariba *(amended version of 2020 assessment)* . The IUCN Red List of Threatened Species 2021: e.T39917A190420483. United Kingdom: IUCN Red List.

[B77] Oklander L. I. Buss G. Bicca-Marques J. C. Hirano Z. Chaves Ó. Mendes S. L. (2022). “Brown howler monkey *Alouatta guariba* (Humboldt, 1812) Brazil, Argentina,” in Primates in peril. The world’s 25 most endangered primates 2022–2023. Editors Mittermeier. R. A. Reuter K. E. Rylands A. B. Jerusalinsky L. Schwitzer C. Strier K. B. (Washington, DC, USA and Bristol UK: IUCN SSC Primate Specialist Group, International Primatological Society, Global Wildlife Conservation, and Bristol Zoological Society), 121–125.

[B78] Oklander L. I. Jerusalinsky L. Bonatto S. L. (2007b). “Applicability of microsatellite heterologs in *Alouatta guariba* and other Brazilian primates,” in Resumos XII Congresso Brasileiro de Primatologia. Editors Melo F. R. Hirsch A. Costa C. G. Dias L. G. Mourthé I. M. C. Tabacow F. P. (Belo Horizonte, Brazil: Sociedade Brasileira de Primatologia), 195.

[B79] Oklander L. I. Kowalewski M. M. Corach D. (2010). Genetic consequences of habitat fragmentation in Black-and-Gold Howler (*Alouatta caraya*) populations from Northern Argentina. Int. J. Primatol. 31, 813–832. 10.1007/s10764-010-9430-6

[B80] Oklander L. I. Marino M. Zunino G. E. Corach D. (2004). Preservation and extraction of DNAfrom feces in howler monkeys (*Alouatta caraya*). Neotrop. Primates 12, 59–63. 10.62015/np.2004.v12.571

[B81] Oklander L. I. Miño C. I. Fernández G. Caputo M. Corach D. (2017). Genetic structure in the southernmost populations of black-and-gold howler monkeys (*Alouatta caraya*) and its conservation implications. PLoS One 12, e0185867. 10.1371/journal.pone.0185867 28968440 PMC5624639

[B82] Oklander L. I. Rheingantz M. Rossato R. S. Peker S. Hirano Z. M. Monticelli C. (2024). Restoration of *Alouatta guariba* populations: building a binational management strategy for the conservation of the Endangered brown howler monkey of the Atlantic Forest. Front. Cons. Sci. 5, 1401749. 10.3389/fcosc.2024.1401749

[B83] Oklander L. I. Zunino G. E. Di Fiore A. Corach D. (2007a). Isolation, characterization and evaluation of 11 autosomal STRs suitable for population studies in black and gold howler monkeys *Alouatta caraya* . Mol. Ecol. Notes 7, 117–120. 10.1111/j.1471-8286.2006.01529.x

[B84] Oliveira E. H. Neusser M. Figueiredo W. B. Nagamachi C. Pieczarka J. C. Sbalqueiro I. J. (2002). The phylogeny of howler monkeys (*Alouatta*, Platyrrhini): reconstruction by multicolor cross-species chromosome painting. Chromosome Res. 10, 669–683. 10.1023/a:1021520529952 12575795

[B85] Oliveira E. H. C. Suemitsu E. Silva A. Sbalqueiro I. J. (2000). Geographical variation of chromosomal number in *Alouatta fusca clamitans* (Primates, Atelidae). Caryologia 53 (2), 163–168. 10.1080/00087114.2000.10589192

[B86] Peakall R. Ruibal M. Lindenmayer D. B. (2003). Spatial autocorrelation analysis offers new insights into gene flow in the Australian bush rat, Rattus fuscipes. Evolution 57, 1182–1195. 10.1111/j.0014-3820.2003.tb00327.x 12836834

[B87] Peakall R. Smouse P. E. (2012). GenAlEx 6.5: genetic analysis in Excel. Population genetic software for teaching and research–an update. Bioinformatics 28, 2537–2539. 10.1093/bioinformatics/bts460 22820204 PMC3463245

[B88] Pessenda L. C. R. Oliveira P. E. Mofatto M. Medeiros V. B. Garcia R. J. F. Aravena R. (2009). The evolution of a tropical rainforest/grassland mosaic in southeastern Brazil since 28,000 ^14^C yr BP based on carbon isotopes and pollen recors. Quat. Res. 71, 427–452.

[B89] Povill C. de Oliveira M. B. de Abreu F. V. S. de Oliveira R. L. Perini F. A. Monticelli C. (2023). Genetic diversity and insights about distribution of brown howler monkeys (*Alouatta guariba* group) (Atelidae, Alouattinae). Int. J. Primatol. 44 (3), 517–539. 10.1007/s10764-023-00352-z

[B90] Pritchard J. K. Stephens M. Donnelly P. (2000). Inference of population structure using multilocus genotype data. Genetics 155, 945–959. 10.1093/genetics/155.2.945 10835412 PMC1461096

[B91] Rambaut A. (2018). FigTree. Version 1.4.4. Available at: http://tree.bio.ed.ac.uk/software-/figtree/.

[B92] Rambaut A. Drummond A. J. Xie D. Baele G. Suchard M. A. (2018). Posterior summarization in Bayesian phylogenetics using Tracer 1.7. Syst. Biol. 675, 901–904. 10.1093/sysbio/syy032 PMC6101584 29718447

[B93] Rozas J. Ferrer-Mata A. Sánchez-DelBarrio J. C. Guirao-Rico S. Librado P. Ramos-Onsins S. E. (2017). DnaSP 6: DNA sequence polymorphism analysis of large data sets. Mol. Biol. Evol. 34, 3299–3302. 10.1093/molbev/msx248 29029172

[B94] Rylands A. B. Brandon-Jones D. (1998). Scientific nomenclature of the red howlers from the northeastern Amazon in Brazil, Venezuela, and the Guianas. Int. J. Primatol. 19 (5), 879–905. 10.1023/a:1020349514553

[B95] Rylands A. B. Fonseca G. A. B. da Leite Y. L. R. Mittermeier R. A. (1996). “Primates of the Atlantic Forest: origin, endemism, distributions and communities,” in Adaptive radiations of the neotropical primates. Editors Norconk M. A. Rosenberger A. L. Garber P. A. (New York: Plenum Press), 21–51.

[B96] Rylands A. B. Mittermeier R. A. Rodríguez-Luna E. (1995). A species list for the New World primates (Platyrrhini): distribution by country, endemism, and conservation status according to the Mace-Land system. Neotrop. Prim. 3, 113–160. 10.62015/np.1995.v3.295

[B97] Rylands A. B. Schneider H. Langguth A. Mittermeier R. A. Groves C. P. Rodríguez-Luna E. (2000). An assessment of the diversity of New World primates. Neotrop. Primates 8 (2), 61–93. 10.62015/np.2000.v8.453

[B98] Rylands A. B. Spironello W. R. Tornisielo V. L. Sá R. L. Kierulff M. C. M. Santos I. B. (1988). Primates of the rio Jequitinhonha valley, Minas Gerais, Brazil. Primate Conserv. 9, 100–119.

[B99] Sambrook J. Fritch E. F. Maniatis T. (1989). Molecular cloning – a laboratory Manual. 2nd edition. New York: Cold Spring Harbor Laboratory Press.

[B100] Santos C. Montiel R. Sierra B. Bettencourt C. Fernandez E. Alvarez L. (2005). Understanding differences between phylogenetic and pedigree-derived mtDNA mutation rate: a model using families from the Azores Islands (Portugal). Mol. Biol. Evol. 22, 1490–1505. 10.1093/molbev/msi141 15814829

[B101] Schrago C. G. (2007). On the time scale of New World primate diversification. Am. J. Phys. Anthropol. 132 (3), 344–354. 10.1002/ajpa.20459 17133436

[B102] Schuelke M. (2000). An economic method for the fluorescent labeling of PCR fragments. Nat. Biotechnol. 18, 233–234. 10.1038/72708 10657137

[B118] Steinberg E. R. Fortes V. B. Rossi L. F. Murer L. Lovato M. Merani M. S. (2017). Cytogenetic characterization of brown howler monkeys, *Alouatta guariba clamitans* (Atelidae, Platyrrhini): meiotic confirmation of an X_1_X_1_X_2_X_2_X_3_X_3_/X_1_X_2_X_3_Y_1_Y_2_ sex chromosome system. Cytogenet. Genome Res. 151, 131–140. 10.1159/000464375 28402969

[B103] Swofford D. L. (2003). Phylogenetic analysis using parsimony (and other methods), version 4b. Sunderland, MA: Sinauer Associates.

[B104] Tajima F. (1989). The effect of change in population size on DNA polymorphism. Genetics 123 (3), 597–601. 10.1093/genetics/123.3.597 2599369 PMC1203832

[B105] Tchaicka l. Eizirik E. de Oliveira T. G. Cândido J. F. Freitas T. R. O. (2007). Phylogeography and population history of the crab-eating fox (*Cerdocyon thous*). Mol. Ecol. 16, 819–838. 10.1111/j.1365-294X.2006.03185.x 17284214

[B106] Thoisy B. Silva A. G. Ruiz-García M. Tapia A. Ramirez O. Arana M. (2010). Population history, phylogeography, and conservation genetics of the last Neotropical mega-herbivore,the lowland tapir (*Tapirus terrestris*). BMC Evol. Biol. 10, 278–293. 10.1186/1471-2148-10-278 20840756 PMC2949869

[B107] Thomas O. (1913). New mammals from South America. Ann. Mag. Nat. Hist. 8 (12), 567–574.

[B108] Thomé M. T. C. Zamudio K. R. Giovanelli J. G. R. Haddad C. F. B. Baldissera F. A. Jr. Alexandrino J. M. B. (2010). Phylogeography of endemic toads and post-Pliocene persistence of the Brazilian Atlantic Forest. Mol. Phylogenet. Evol. 55, 1018–1031. 10.1016/j.ympev.2010.02.003 20139019

[B109] Tomer Y. Greenberg D. A. Concepción E. Ban Y. Davies T. F. (2002). Thyroglobulin is a thyroid specific gene for the familial autoimmune Thyroid diseases. J. Clin. Endocrinol. Metab. 87, 404–407. 10.1210/jcem.87.1.8291 11788684

[B110] Urbani B. Cormier L. A. (2015). “The ethnoprimatology of the howler monkeys (*Alouatta* spp.): from past to present,” in Howler monkeys: behavior, ecology, and conservation. Editors Kowalewski M. M. Garber P. A. Cortés-Ortiz L. Urbani B. Youlatos D. (New York: Springer), 259–280. 10.1007/978-1-4939-1960-4

[B111] Van Oosterhout C. Hutchinson W. F. Wills D. P. M. Shipley P. (2004). MICRO-CHECKER: software for identifying and correcting genotyping errors in microsatellite data. Mol. Ecol. Notes 4, 535–538. 10.1111/j.1471-8286.2004.00684.x

[B112] Vieira C. da C. (1944). Os símios do estado de São Paulo. Pap. Avuls. Zool., São Paulo 4, 1–31. 10.11606/0031-1049.1944.4p1-31

[B113] Wang J. (2019). A parsimony estimator of the number of populations from a STRUCTURE-like analysis. Mol. Ecol. Resour. 19, 970–981. 10.1111/1755-0998.13000 30681281

[B114] Weir B. S. Cockerham C. C. (1984). Estimating F-statistics for the analysis of population structure. Evolution 38 (6), 1358–1370. 10.1111/j.1558-5646.1984.tb05657.x 28563791

[B115] Woodburne M. O. (2010). The great American biotic interchange: dispersals, tectonics, climate, sea level and holding pens. J. Mamm. Evol. 17, 245–264. 10.1007/s10914-010-9144-8 21125025 PMC2987556

[B116] Xia X. Xie Z. (2001). DAMBE: software package for data analysis in molecular biology and evolution. J. Hered. 92 (4), 371–373. 10.1093/jhered/92.4.371 11535656

[B117] Xia X. Xie Z. Salemi M. Chen L. Wang Y. (2003). An index of substitution saturation and its application. Mol. Phylogenet. Evol. 26, 1–7. 10.1016/S1055-7903(02)00326-3 12470932

