# Supplementary material for: Phylogeography, taxonomy, and conservation of the endangered brown howler monkey, Alouatta guariba (Primates, Atelidae), of the Atlantic Forest
Source: Front Genet. 2024 Dec 3;15:1453005. doi: 10.3389/fgene.2024.1453005 (PMC11683736; doi:10.3389/fgene.2024.1453005)
Supplement: Supplementary file 1 [file DataSheet4.docx]

**Phylogeography, Taxonomy and Conservation of the Endangered Brown Howler Monkey, *Alouatta guariba* (Primates, Atelidae), of the Atlantic Forest.**

**Supplementary Material 4: Extra Figures of Microsatellite Results**

**Figure of TESS for K=2**

**
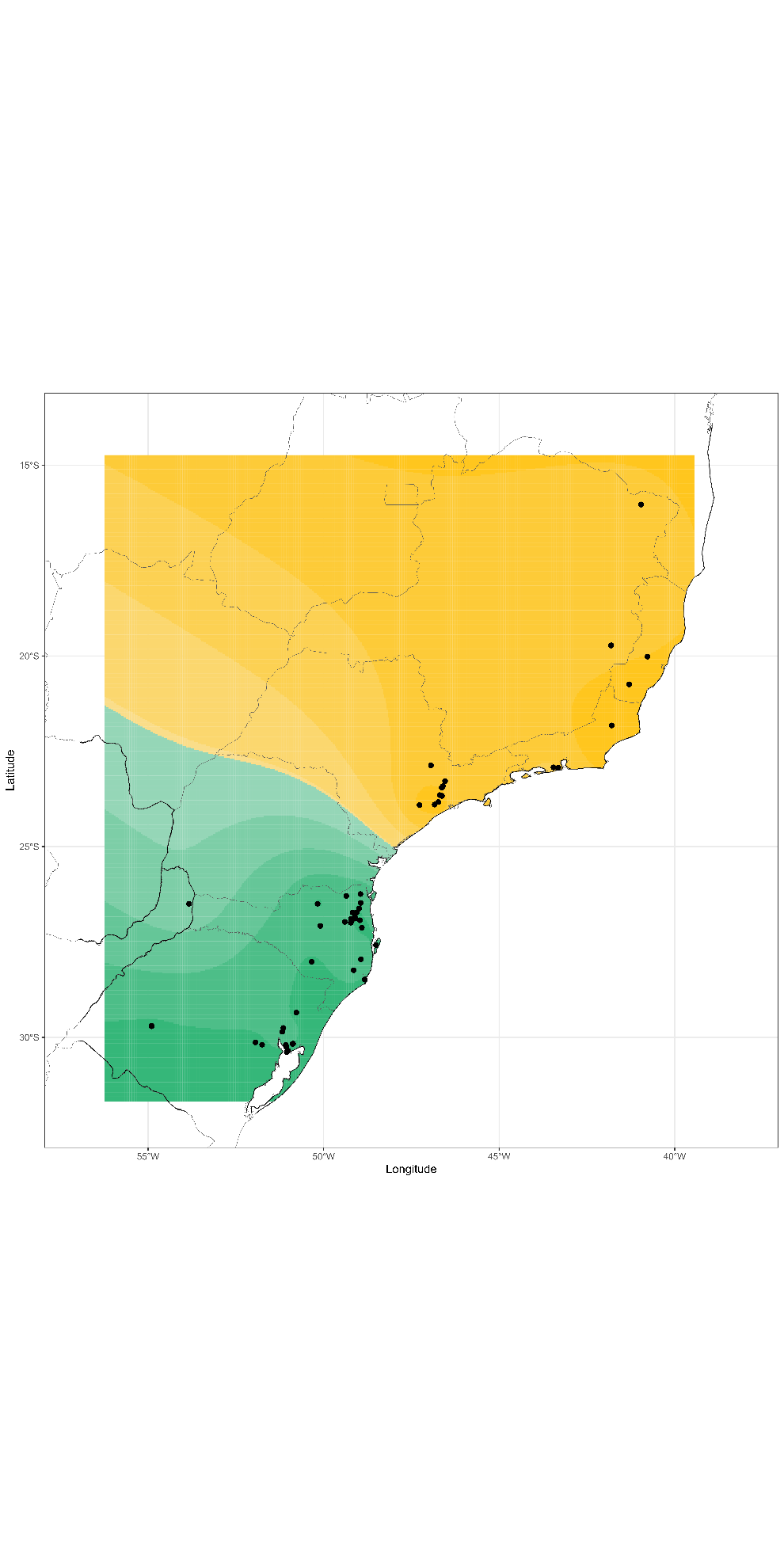
**

**Figure of Principal Component Analysis**

**
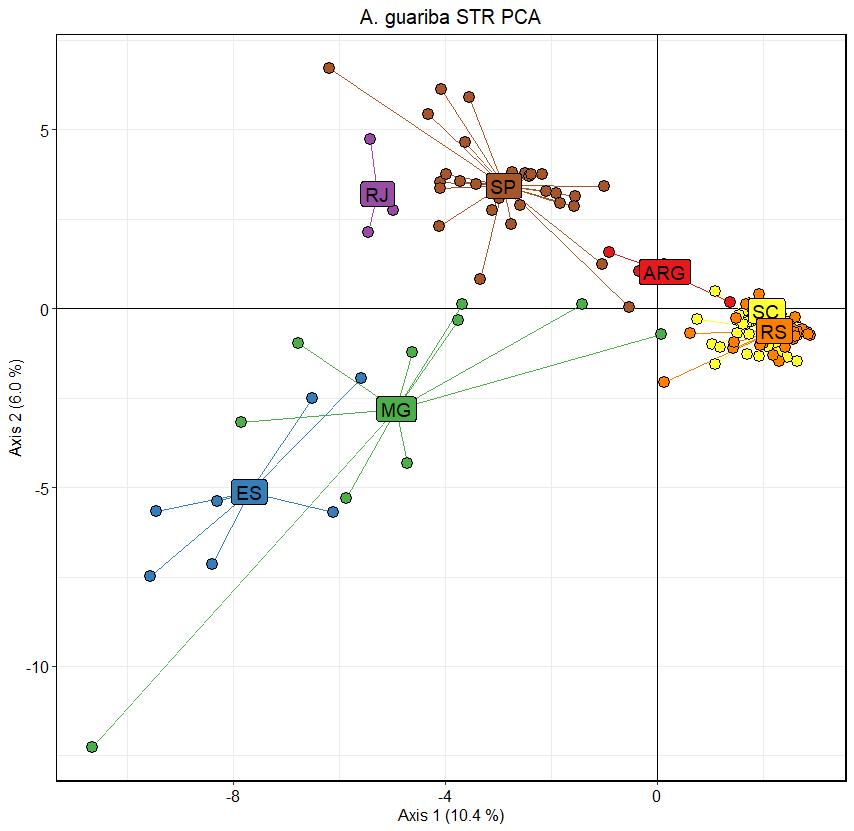
**

**Figure of FSTs**

**
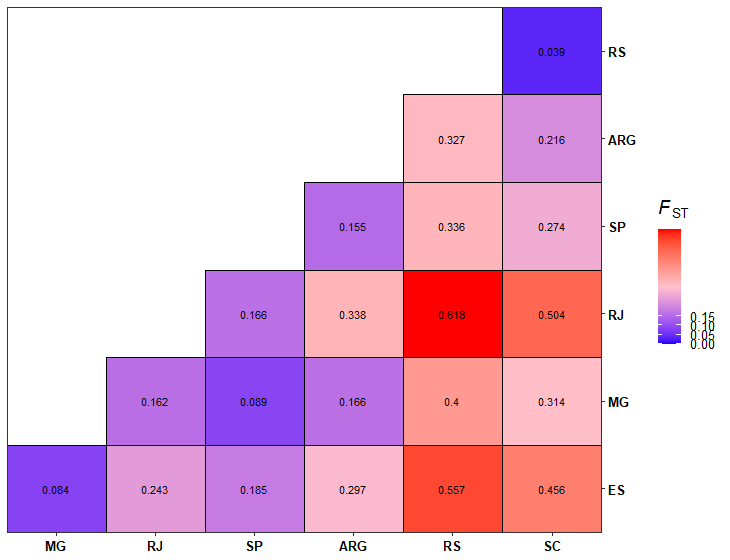
**
