# Supplementary material for: Phylogeography, taxonomy, and conservation of the endangered brown howler monkey, Alouatta guariba (Primates, Atelidae), of the Atlantic Forest
Source: Front Genet. 2024 Dec 3;15:1453005. doi: 10.3389/fgene.2024.1453005 (PMC11683736; doi:10.3389/fgene.2024.1453005)
Supplement: Supplementary file 2 [file DataSheet3.docx]

**Phylogeography, Taxonomy and Conservation of the Endangered Brown Howler Monkey, *Alouatta guariba* (Primates, Atelidae), of the Atlantic Forest.**

**Supplementary Material 3. Data on 10 polymorphic loci in the studied populations**

| **PopName** | **Locus** | **N** | **Na** | **Ne** | **Ho** | **He** | **uHe** | **Hs** | **F** | **PA** | **pHWE** |
| --- | --- | --- | --- | --- | --- | --- | --- | --- | --- | --- | --- |
| **ES** | **AB17a** | 7 | 4.000 | 2.970 | 0.857 | 0.663 | 0.714 | 0.702 | -0.292 | - | 0.734 |
|  | **AB7a** | 7 | 4.000 | 2.970 | 0.429 | 0.663 | 0.714 | 0.738 | 0.354 | - | 0.234 |
|  | **AB10a** | 7 | 4.000 | 3.630 | 1.000 | 0.724 | 0.780 | 0.762 | -0.380 | 1 | 0.321 |
|  | **AC17a** | 7 | 4.000 | 3.379 | 0.714 | 0.704 | 0.758 | 0.762 | -0.014 | - | 0.358 |
|  | **AC14a** | 7 | 4.000 | 2.579 | 0.714 | 0.612 | 0.659 | 0.655 | -0.167 | - | 0.744 |
|  | **D118a** | 7 | 3.000 | 1.815 | 0.571 | 0.449 | 0.484 | 0.476 | -0.273 | - | 0.772 |
|  | **D157a** |  |  |  |  |  |  |  |  | - |  |
|  | **T1a** | 7 | 5.000 | 3.379 | 0.714 | 0.704 | 0.758 | 0.762 | -0.014 | 1 | 0.085 |
|  | **D17a** |  |  |  |  |  |  |  |  | - |  |
|  | **D8a** | 7 | 3.000 | 2.279 | 0.714 | 0.561 | 0.604 | 0.595 | -0.273 | - | 0.641 |
|  | **Average** |  | 3.875 | 2.875 | 0.714 | 0.635 | 0.684 | 0.682 |  |  |  |
|  | **Std** |  | 0.641 | 0.617 | 0.171 | 0.092 | 0.099 | 0.102 |  |  |  |
| **MG** | **AB17a** | 10 | 5.000 | 4.167 | 0.800 | 0.760 | 0.800 | 0.8 | -0.053 | - | 0.698 |
|  | **AB7a** | 10 | 6.000 | 4.167 | 0.700 | 0.760 | 0.800 | 0.806 | 0.079 | - | 0.457 |
|  | **AB10a** | 10 | 7.000 | 3.846 | 0.800 | 0.740 | 0.779 | 0.778 | -0.081 | 2 | 0.401 |
|  | **AC17a** | 10 | 3.000 | 2.740 | 0.500 | 0.635 | 0.668 | 0.678 | 0.213 | - | 0.324 |
|  | **AC14a** | 10 | 5.000 | 1.942 | 0.500 | 0.485 | 0.511 | 0.511 | -0.031 | - | 0.432 |
|  | **D118a** | 10 | 3.000 | 1.515 | 0.400 | 0.340 | 0.358 | 0.356 | -0.176 | - | 0.891 |
|  | **D157a^c^** | 8 | 6.000 | 4.571 | 1.000 | 0.781 | 0.833 | 0.821 | -0.280 | 2 | 0.065 |
|  | **T1a** | 10 | 5.000 | 2.469 | 0.600 | 0.595 | 0.626 | 0.628 | -0.008 | 1 | 0.206 |
|  | **D17a^c^** | 8 | 5.000 | 3.765 | 0.250 | 0.734 | 0.783 | 0.821 | 0.660 | - | 0.083 |
|  | **D8a** | 10 | 6.000 | 4.082 | 0.700 | 0.755 | 0.795 | 0.8 | 0.073 | 2 | 0.090 |
|  | **Average** |  | 5.100 | 3.326 | 0.625 | 0.659 | 0.695 | 0.700 |  |  |  |
|  | **Std** |  | 1.287 | 1.068 | 0.220 | 0.147 | 0.156 | 0.159 |  |  |  |
| **RJ** | **AB17a** | 3 | 2.000 | 2.000 | 0.333 | 0.500 | 0.600 | 0.667 | 0.333 | - | 0.564 |
|  | **AB7a** | 3 | 3.000 | 2.571 | 0.667 | 0.611 | 0.733 | 0.75 | -0.091 | - | 0.506 |
|  | **AB10a** | 3 | 2.000 | 1.385 | 0.333 | 0.278 | 0.333 | 0.333 | -0.200 | - | 0.729 |
|  | **AC17a** | 3 | 2.000 | 1.385 | 0.333 | 0.278 | 0.333 | 0.333 | -0.200 | - | 0.729 |
|  | **AC14a** | 3 | 2.000 | 1.385 | 0.333 | 0.278 | 0.333 | 0.333 | -0.200 | - | 0.729 |
|  | **D118a** | 3 | 4.000 | 3.600 | 1.000 | 0.722 | 0.867 | 0.833 | -0.385 | 2 | 0.609 |
|  | **D157a** | 3 | 2.000 | 2.000 | 0.333 | 0.500 | 0.600 | 0.667 | 0.333 | - | 0.564 |
|  | **T1a** | 3 | 1.000 | 1.000 | 0.000 | 0.000 | 0.000 | 0 |  | - |  |
|  | **D17a** | 3 | 3.000 | 2.571 | 0.667 | 0.611 | 0.733 | 0.75 | -0.091 | - | 0.506 |
|  | **D8a** | 3 | 2.000 | 2.000 | 0.333 | 0.500 | 0.600 | 0.667 | 0.333 | - | 0.564 |
|  | **Average** |  | 2.300 | 1.990 | 0.433 | 0.428 | 0.513 | 0.533 |  |  |  |
|  | **Std** |  | 0.823 | 0.771 | 0.274 | 0.216 | 0.259 | 0.267 |  |  |  |
| **SP** | **AB17a^a^** | 29 | 10.000 | 5.160 | 0.897 | 0.806 | 0.820 | 0.819 | -0.112 | 2 | 0.751 |
|  | **AB7a** | 31 | 10.000 | 5.445 | 0.774 | 0.816 | 0.830 | 0.831 | 0.052 | 1 | 0.111 |
|  | **AB10a** | 31 | 3.000 | 1.684 | 0.355 | 0.406 | 0.413 | 0.414 | 0.127 | - | 0.790 |
|  | **AC17a** | 31 | 5.000 | 3.056 | 0.548 | 0.673 | 0.684 | 0.686 | 0.185 | 1 | 0.290 |
|  | **AC14a^a^** | 29 | 3.000 | 2.059 | 0.448 | 0.514 | 0.523 | 0.525 | 0.128 | - | 0.397 |
|  | **D118a^a^** | 28 | 8.000 | 3.200 | 0.571 | 0.688 | 0.700 | 0.702 | 0.169 | 3 | 0.070 |
|  | **D157a^b^** | 26 | 7.000 | 4.492 | 0.808 | 0.777 | 0.793 | 0.792 | -0.039 | - | 0.902 |
|  | **T1a** | 31 | 2.000 | 1.067 | 0.065 | 0.062 | 0.063 | 0.063 | -0.033 | - | 0.853 |
|  | **D17a** | 31 | 10.000 | 4.587 | 0.677 | 0.782 | 0.795 | 0.797 | 0.134 | 2 | 0.903 |
|  | **D8a^b^** | 26 | 5.000 | 2.414 | 0.462 | 0.586 | 0.597 | 0.6 | 0.212 | 1 | 0.067 |
|  | **Average** |  | 6.300 | 3.316 | 0.560 | 0.611 | 0.622 | 0.623 |  |  |  |
|  | **Std** |  | 3.129 | 1.532 | 0.246 | 0.236 | 0.240 | 0.240 |  |  |  |
| **SC** | **AB17a** | 59 | 12.000 | 1.918 | 0.458 | 0.479 | 0.483 | 0.483 | 0.044 | 6 | 1.000 |
|  | **AB7a^a^** | 56 | 6.000 | 1.864 | 0.339 | 0.464 | 0.468 | 0.469 | 0.268 | - | 0.386 |
|  | **AB10a** | 60 | 6.000 | 1.250 | 0.217 | 0.200 | 0.202 | 0.202 | -0.083 | 2 | 1.000 |
|  | **AC17a** | 59 | 3.000 | 1.419 | 0.237 | 0.295 | 0.298 | 0.299 | 0.197 | 1 | 0.081 |
|  | **AC14a** | 60 | 2.000 | 1.069 | 0.067 | 0.064 | 0.065 | 0.065 | -0.034 | - | 0.789 |
|  | **D118a** | 60 | 3.000 | 1.034 | 0.033 | 0.033 | 0.033 | 0.033 | -0.013 | - | 0.999 |
|  | **D157a** | 59 | 9.000 | 3.782 | 0.746 | 0.736 | 0.742 | 0.742 | -0.014 | 2 | 0.982 |
|  | **T1a** | 57 | 3.000 | 1.054 | 0.053 | 0.052 | 0.052 | 0.052 | -0.021 | 1 | 0.998 |
|  | **D17a** | 58 | 4.000 | 1.459 | 0.293 | 0.315 | 0.317 | 0.318 | 0.068 | - | 0.538 |
|  | **D8a** | 60 | 6.000 | 1.793 | 0.467 | 0.442 | 0.446 | 0.446 | -0.055 | 2 | 0.250 |
|  | **Average** |  | 5.400 | 1.664 | 0.291 | 0.308 | 0.311 | 0.311 |  |  |  |
|  | **Std** |  | 3.134 | 0.817 | 0.224 | 0.228 | 0.230 | 0.230 |  |  |  |
| **ARG** | **AB17a** | 5 | 2.000 | 2.000 | 0.600 | 0.500 | 0.556 | 0.55 | -0.200 | - | 0.655 |
|  | **AB7a** | 5 | 2.000 | 1.220 | 0.200 | 0.180 | 0.200 | 0.2 | -0.111 | - | 0.804 |
|  | **AB10a** | 5 | 2.000 | 1.724 | 0.200 | 0.420 | 0.467 | 0.5 | 0.524 | - | 0.241 |
|  | **AC17a** | 5 | 3.000 | 2.632 | 0.600 | 0.620 | 0.689 | 0.7 | 0.032 | - | 0.137 |
|  | **AC14a** | 5 | 1.000 | 1.000 | 0.000 | 0.000 | 0.000 | 0 |  | - |  |
|  | **D118a** | 5 | 2.000 | 1.724 | 0.200 | 0.420 | 0.467 | 0.5 | 0.524 | - | 0.241 |
|  | **D157a** | 5 | 4.000 | 3.571 | 1.000 | 0.720 | 0.800 | 0.775 | -0.389 | - | 0.544 |
|  | **T1a** | 5 | 1.000 | 1.000 | 0.000 | 0.000 | 0.000 | 0 |  | - |  |
|  | **D17a** | 5 | 3.000 | 2.381 | 0.800 | 0.580 | 0.644 | 0.625 | -0.379 | - | 0.423 |
|  | **D8a** | 5 | 3.000 | 2.174 | 0.800 | 0.540 | 0.600 | 0.575 | -0.481 | - | 0.528 |
|  | **Average** |  | 2.300 | 1.943 | 0.440 | 0.398 | 0.442 | 0.443 |  |  |  |
|  | **Std** |  | 0.949 | 0.801 | 0.363 | 0.254 | 0.283 | 0.278 |  |  |  |
| **RS** | **AB17a** | 38 | 4.000 | 1.345 | 0.184 | 0.257 | 0.260 | 0.261 | 0.282 | - | 0.269 |
|  | **AB7a** | 38 | 3.000 | 1.083 | 0.079 | 0.077 | 0.078 | 0.078 | -0.032 | - | 0.996 |
|  | **AB10a** | 38 | 2.000 | 1.027 | 0.026 | 0.026 | 0.026 | 0.026 | -0.013 | - | 0.934 |
|  | **AC17a^b^** | 32 | 2.000 | 1.168 | 0.156 | 0.144 | 0.146 | 0.146 | -0.085 | - | 0.632 |
|  | **AC14a** | 38 | 2.000 | 1.082 | 0.079 | 0.076 | 0.077 | 0.077 | -0.041 | - | 0.800 |
|  | **D118a** | 38 | 3.000 | 1.112 | 0.105 | 0.101 | 0.102 | 0.102 | -0.041 | 1 | 0.990 |
|  | **D157a^c^** | 29 | 6.000 | 2.572 | 0.517 | 0.611 | 0.622 | 0.624 | 0.154 | 1 | 0.964 |
|  | **T1a^a^** | 35 | 2.000 | 1.153 | 0.143 | 0.133 | 0.135 | 0.134 | -0.077 | - | 0.649 |
|  | **D17a^c^** | 27 | 6.000 | 2.000 | 0.444 | 0.500 | 0.509 | 0.511 | 0.111 | 1 | 0.129 |
|  | **D8a^a^** | 36 | 2.000 | 1.180 | 0.111 | 0.153 | 0.155 | 0.156 | 0.273 | - | 0.102 |
|  | **Average** |  | 3.200 | 1.372 | 0.185 | 0.208 | 0.211 | 0.212 |  |  |  |
|  | **Std** |  | 1.619 | 0.507 | 0.163 | 0.195 | 0.199 | 0.199 |  |  |  |

Espírito Santo (ES); Minas Gerais (MG); Rio de Janeiro (RJ); São Paulo (SP); Santa Catarina (SC); Rio Grande do Sul (RS); and the Argentine population (ARG). Na = N° of different alleles; Ne = N° of effective alleles (calculated as 1/Σ (allele frequency)^2^); He = expected heterozygosity = 1 - Σ (allele frequency)^2^; uHe = unbiased expected heterozygosity = (2N / (2N-1)) * He;(Hs): Gene diversity; F = Fixation Index = (He - Ho) / He = 1 - (Ho / He); PA = N° of alleles unique to a single population.^a^missing data <10%;^b^ missing data 10-20%;^c^ missing data 20-30%.
