# Supplementary material for: Phylogeography, taxonomy, and conservation of the endangered brown howler monkey, Alouatta guariba (Primates, Atelidae), of the Atlantic Forest
Source: Front Genet. 2024 Dec 3;15:1453005. doi: 10.3389/fgene.2024.1453005 (PMC11683736; doi:10.3389/fgene.2024.1453005)

**Phylogeography, Taxonomy and Conservation of the Endangered Brown Howler Monkey, *Alouatta guariba* (Primates, Atelidae), of the Atlantic Forest.**

**Supplementary Material 5. Extra Figures of Mitochondrial Markers Results**

**Figure SF5. 1.** Bayesian phylogenetic tree with divergence times using both mtDNA CR plus CytB sequences (N=109 seq; 1274pb). Clades labels are above the branches, and below the Posterior Probability. Bottom rule is divergence time in years.

**Figure SF5. 2**. ML phylogenetic tree from the 116 mtDNA CytB sequences of *A. guariba* obtained in this study (N=17 haplotypes; 678pb) and other 25 *A. guariba* haplotypes (Povill et al. 2023) retrieved from the GenBank. Main phylogenetic clades (A, B and C) are identified.

0.02

**Figure SF5. 3**. Genetic clusters obtained for mtDNA control region by BAPS (best K = 6, Log marginal likelihood = -4269.9023). Each cluster is represented by a color.


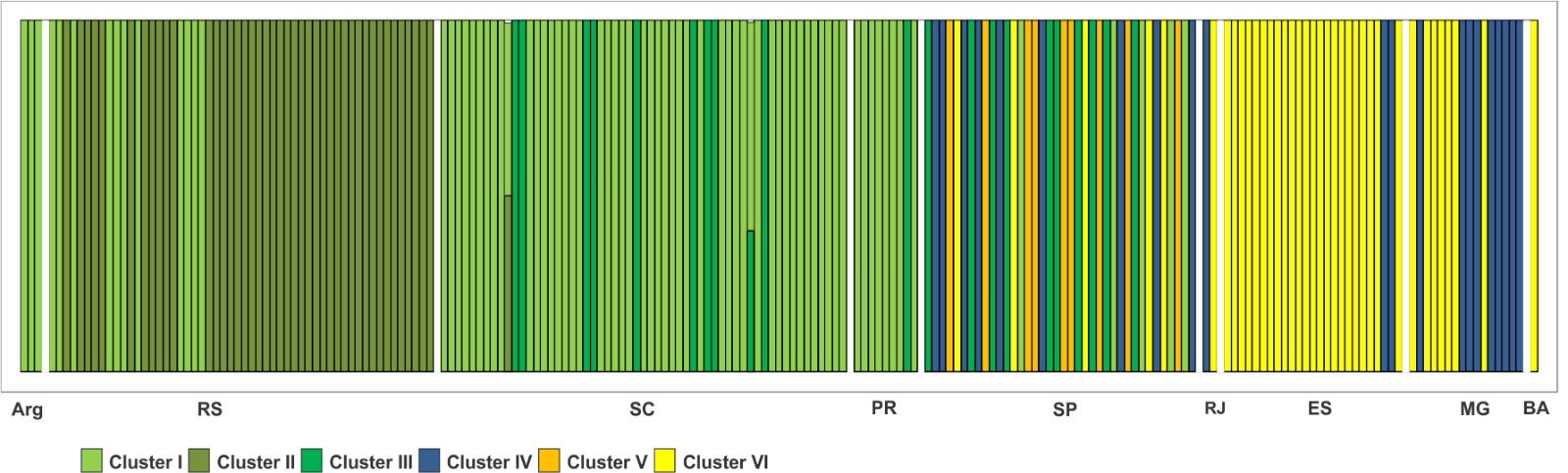

Supplement: Supplementary file 3 [file DataSheet5.docx]
