# Supplementary material for: Phylogeography, taxonomy, and conservation of the endangered brown howler monkey, Alouatta guariba (Primates, Atelidae), of the Atlantic Forest
Source: Front Genet. 2024 Dec 3;15:1453005. doi: 10.3389/fgene.2024.1453005 (PMC11683736; doi:10.3389/fgene.2024.1453005)
Supplement: Supplementary file 4 [file Table2.DOCX]

**Phylogeography of the endangered brown howler monkey, *Alouatta guariba* (Primates, Atelidae) of the Atlantic Forest: evolution, taxonomy, and conservation.**

**Supplementary File 2**

**Table SF2.1**. Sequences used for estimate CytB rate in Platyrrhini superfamily.

| **Family** | **Genus** | **Species** | **ID GenBank** |
| --- | --- | --- | --- |
|  |  |  |  |
| **Pitheciidae** | *Callicebus* | *C. hoffmannsi* | AF524885.1 |
|  |  | *C. lugens* | AF524888.2 |
|  |  | *C. moloch* | AF524887.1 |
|  |  | *C. torquatus* | AF524890.2 |
|  | *Pithecia* | *P. irrorata* | AY226183.1 |
|  |  | *P. monachus* | FJ531668.1 |
|  | *Chiropotes* | *C. chiropotes* | FJ531667.1 |
|  |  | *C. israelita* | AY226187.1 |
|  |  | *C. utahicki* | AY226186.1 |
|  | *Cacajao* | *C. calvus* | FJ531664.1 |
|  |  | *C. melanocephalus* | FJ531646.1 |
|  |  |  |  |
| **Cebidae** | *Cebus* | *C. albifrons* | FJ529108.1 |
|  |  | *C. apella* | FJ529103.1 |
|  |  | *C. capucinus* | AY065907.1 |
|  |  | *C. cay* | FJ529088.1 |
|  |  | *C. olivaceus* | FJ529107.1 |
|  | *Saimiri* | *S. boliviensis* | AJ315388.1 |
|  |  | *S. oerstedii* | EU232702.1 |
|  |  | *S. sciureus* | AJ489747.1 |
|  |  | *S. ustus* | EU232707.1 |
|  | *Aotus* | *A. azarai* | DQ098865.1 |
|  |  | *A. lemurinus* | DQ098871.1 |
|  |  | *A. nancymaae* | AJ489746.1 |
|  |  | *A. trivirgatus* | DQ098874.1 |
|  | *Saguinus* | *S. midas* | AJ489760.1 |
|  | *Callithrix* | *C. jacchus* | AF295586.1 |
|  |  |  |  |
| **Atelidae** | *Alouatta* | *A. belzebul* | DQ387015.1 |
|  |  | *A. macconnelli* | AY065888.1 |
|  |  | *A. palliata* | AY065878.1 |
|  |  | *A. pigra* | AY065884.1 |
|  |  | *A. sara* | AY065887.1 |
|  |  | *A. seniculus* | AJ489759.1 |
|  | *Ateles* | *A. belzebuth* | FJ785422.1 |
|  |  | *A. geoffroyi* | AY065903.1 |
|  | *Brachyteles* | *B. arachnoides* | AY065905.1 |
|  | *Lagothrix* | *L. lagotricha* | AY671799.1 |
|  |  |  |  |

**Table SF2.2**. Sequences of mitochondrial CytB gene of the genus *Alouatta* and genus *Ateles* (outgroups) retrieved from GenBank and used for estimate the phylogenetic relationship among *A. guariba* lineage.

| **Species** | **ID Genbank** | **Reference** |
| --- | --- | --- |
| *Alouatta guariba* | [OP985658-985779] | Povill et al. 2023 |
|  | DQ679775 | Harris et al. 2005 |
|  | DQ679779 | Harris et al. 2005 |
|  | HQ385485 | Martins et al. 2011 |
|  | HQ385488 | Martins et al. 2011 |
| *A. caraya* | DQ350637 | Harris et al. 2005 |
| *A. belzebul* | DQ387044 | Harris et al. 2005 |
| *A. seniculus* | EU232713 | Lavergne et al. 2010 |
| *A. s. macconnelli* | AJ489759 | Lavergne et al. 2010 |
| *A. sara* | AY065887 | Cortés-Ortiz et al., 2003 |
| *A. pigra* | AY065885 | Cortés-Ortiz et al., 2003 |
| *A. paliatta* | AY065880 | Cortés-Ortiz et al., 2003 |
| *Ateles geoffroyi*  *Ateles belzebuth* | KR902382  KR902369 | Ghersi et al. 2015  Ghersi et al. 2015 |

**Table SF2.3**. Haplotypes (Hap) of mitochondrial CytB gene (678pb) obtained in this study for each sample of *A. guariba*. Clades (A, B, C), GenBank accession number (ID GenBank) and sampling sites (Country, State/Province) are shown.

| **Hap** | **ID Sample** | **Clade** | **ID GenBank** | **Country, State/Province** |
| --- | --- | --- | --- | --- |
| H1 | AG207, L18 | A | PQ278407 | Brazil, Minas Gerais (MG) |
| H1 | AG189, AG191, AG192, AG193, AG195, AG196, AG197 | A | PQ278407 | Brazil, Espírito Santo (ES) |
| H1 | AG8, AG16, AG39, AG42 | A | PQ278407 | Brazil, São Paulo (SP) |
| H2 | AG201, AG206 | B | PQ278408 | Brazil, Minas Gerais (MG) |
| H2 | AG2, AG4, AG9, AG11, AG20, AG35, AG40, AG41, L16 | B | PQ278408 | Brazil, São Paulo (SP) |
| H3 | AG198 | A | PQ278409 | Brazil, Espírito Santo (ES) |
| H4 | S61 | C | PQ278410 | Brazil, Santa Catarina (SC) |
| H4 | AG1, AG3, AG10, AG13, AG21, AG22, AG33, AG37 | C | PQ278410 | Brazil, São Paulo (SP) |
| H5 | L19 | B | PQ278411 | Brazil, Minas Gerais (MG) |
| H5 | AG5 | B | PQ278411 | Brazil, São Paulo (SP) |
| H6 | AG7, AG12, AG18, AG19, AG32, AG36, AG44 | A | PQ278412 | Brazil, São Paulo (SP) |
| H7 | AG17 | C | PQ278413 | Brazil, São Paulo (SP) |
| H8 | AG29 |  | PQ278414 | Brazil, Rio de Janeiro (RJ) |
| H9 | AG30 |  | PQ278415 | Brazil, São Paulo (SP) |
| H10 | AG31 |  | PQ278416 | Brazil, São Paulo (SP) |
| H11 | AG34, AG43, L22 | C | PQ278417 | Brazil, São Paulo (SP) |
| H11 | L24, L25, L26, L29, L30, S50, S51, S52, S53, S54, S55, S56, S57, S58, S59, S62, S64, S65, S66, S67, S68, S69, S73, S75, S77, S78, S80, S81, S83, S86, S87, S88, S91, S92, S93, S94, S96, S97, S98, S101, S102, S103, S104, S106, S107, S108, S110 | C | PQ278417 | Brazil, Santa Catarina (SC) |
| H11 | L4, L40, L42, L43, Rabo, X5, X7 | C | PQ278417 | Brazil, Rio Grande do Sul (RS) |
| H11 | PG3 | C | PQ278417 | Argentina, Misiones (ARG) |
| H12 | AG38 | C | PQ278418 | Brazil, São Paulo (SP) |
| H13 | L31, X6 | C | PQ278419 | Brazil, Rio Grande do Sul (RS) |
| H14 | S60, S72, S95, S105 | C | PQ278420 | Brazil, Santa Catarina (SC) |
| H15 | S76 | B | PQ278421 | Brazil, Rio de Janeiro (RJ) |
| H16 | S99 | C | PQ278422 | Brazil, Santa Catarina (SC) |
| H17 | X9 | C | PQ278423 | Brazil, Rio Grande do Sul (RS) |

**Table SF2.4.** Haplotypes (Hap) of mitochondrial Control Region (596 pb) obtained in this study for each sample of *A. guariba*. Additionally, clades (A, B, C), GenBank accession number (ID GenBank) and sampling sites (Country, State/Province) are shown.

| **Hap** | **ID Sample** | **Clade** | **ID GenBank** | **Country, State/Province** |
| --- | --- | --- | --- | --- |
| H1 | A.g.g.11 | A | PQ278424 | Brazil, Espírito Santo (ES) |
| H2 | A.g.g.12, A.g.g.13 | B | PQ278425 | Brazil, Espírito Santo (ES) |
| H3 | A.g.g.17 | A | PQ278426 | Brazil, Espírito Santo (ES) |
| H4 | BG20, BG21, BG22 | B | PQ278427 | Brazil, Minas Gerais (MG) |
| H5 | BG9, BG10 | A | PQ278428 | Brazil, Espírito Santo (ES) |
| H6 | AG202, AG207 | A | PQ278429 | Brazil, Minas Gerais (MG) |
| H7 | AG203, AG205, AG206 | B | PQ278430 | Brazil, Minas Gerais (MG) |
| H8 | AG1, AG33, AG37 | C | PQ278431 | Brazil, São Paulo (SP) |
| H9 | AG2, AG14, AG40 | B | PQ278432 | Brazil, São Paulo (SP) |
| H10 | AG3, AG21 | C | PQ278433 | Brazil, São Paulo (SP) |
| H11 | AG4 | B | PQ278434 | Brazil, São Paulo (SP) |
| H12 | AG5 | B | PQ278435 | Brazil, São Paulo (SP) |
| H13 | AG7, AG19, AG23, AG25 | A | PQ278436 | Brazil, São Paulo (SP) |
| H14 | AG8, AG16, AG39 | A | PQ278437 | Brazil, São Paulo (SP) |
| H15 | AG9 | B | PQ278438 | Brazil, São Paulo (SP) |
| H16 | AG10 | A | PQ278439 | Brazil, São Paulo (SP) |
| H17 | AG11 | B | PQ278440 | Brazil, São Paulo (SP) |
| H18 | AG12 | A | PQ278441 | Brazil, São Paulo (SP) |
| H19 | AG13 | C | PQ278442 | Brazil, São Paulo (SP) |
| H20 | AG15, AG22 | C | PQ278443 | Brazil, São Paulo (SP) |
| H21 | AG17 | C | PQ278444 | Brazil, São Paulo (SP) |
| H22 | AG18 | A | PQ278445 | Brazil, São Paulo (SP) |
| H23 | AG20 | B | PQ278446 | Brazil, São Paulo (SP) |
| H24 | AG27 | C | PQ278447 | Brazil, São Paulo (SP) |
| H25 | AG29 | B | PQ278448 | Brazil, Rio de Janeiro (RJ) |
| H26 | AG30 | A | PQ278449 | Brazil, São Paulo (SP) |
| H27 | AG31 | C | PQ278450 | Brazil, São Paulo (SP) |
| H28 | AG32, AG36 | A | PQ278451 | Brazil, São Paulo (SP) |
| H29 | AG34 | C | PQ278452 | Brazil, São Paulo (SP) |
| H30 | AG35 | B | PQ278453 | Brazil, São Paulo (SP) |
| H31 | AG38 | C | PQ278454 | Brazil, São Paulo (SP) |
| H32 | AG42 | A | PQ278455 | Brazil, São Paulo (SP) |
| H33 | AG43 | C | PQ278456 | Brazil, São Paulo (SP) |
| H34 | AG44 | A | PQ278457 | Brazil, São Paulo (SP) |
| H35 | AG45, AG46, AG51, AG52, AG54 | A | PQ278458 | Brazil, Minas Gerais (MG) |
| H36 | AG47 | B | PQ278459 | Brazil, Minas Gerais (MG) |
| H37 | AG49, AG50, AG56, AG187 | A | PQ278460 | Brazil, Espírito Santo (ES) |
| H38 | AG53 | A | PQ278461 | Brazil, Minas Gerais (MG) |
| H39 | AG178 | A | PQ278462 | Brazil, Espírito Santo (ES) |
| H40 | AG183, AG186 | A | PQ278463 | Brazil, Espírito Santo (ES) |
| H41 | AG184 | A | PQ278464 | Brazil, Espírito Santo (ES) |
| H42 | AG189, AG190 | A | PQ278465 | Brazil, Espírito Santo (ES) |
| H43 | AG191 | A | PQ278466 | Brazil, Espírito Santo (ES) |
| H44 | AG192, AG193 | A | PQ278467 | Brazil, Espírito Santo (ES) |
| H45 | AG194, AG195 | A | PQ278468 | Brazil, Espírito Santo (ES) |
| H46 | AG196, AG197, AG200 | A | PQ278469 | Brazil, Espírito Santo (ES) |
| H47 | AG198 | A | PQ278470 | Brazil, Espírito Santo (ES) |
| H48 | L2, L20 | C | PQ278471 | Brazil, Rio Grande do Sul (RS) |
| H49 | L4 | C | PQ278472 | Brazil, Rio Grande do Sul (RS) |
|  | L26, S94 | C |  | Brazil, Santa Catarina (SC) |
| H50 | L28, L29, S75 | C | PQ278473 | Brazil, Santa Catarina (SC) |
| H51 | L9, Rabo | C | PQ278474 | Brazil, Rio Grande do Sul (RS) |
| H52 | L13, X8, X7 | C | PQ278475 | Brazil, Rio Grande do Sul (RS) |
| H53 | L19 | B | PQ278476 | Brazil, Minas Gerais (MG) |
| H54 | L22 | C | PQ278477 | Brazil, São Paulo (SP) |
| H55 | L24 | C | PQ278478 | Brazil, Santa Catarina (SC) |
| H56 | L25 | C | PQ278479 | Brazil, Santa Catarina (SC) |
| H57 | L30, S110 | C | PQ278480 | Brazil, Santa Catarina (SC) |
| H58 | L31, L35, L37, L38, X6 | C | PQ278481 | Brazil, Rio Grande do Sul (RS) |
| H59 | L40 | C | PQ278482 | Brazil, Rio Grande do Sul (RS) |
| H60 | L42 | C | PQ278483 | Brazil, Rio Grande do Sul (RS) |
| H61 | L43 | C | PQ278484 | Brazil, Rio Grande do Sul (RS) |
| H62 | L44, L45, L46 | C | PQ278485 | Brazil, Rio Grande do Sul (RS) |
| H63 | S50 | C | PQ278486 | Brazil, Santa Catarina (SC) |
| H64 | S51 | C | PQ278487 | Brazil, Santa Catarina (SC) |
| H65 | S52 | C | PQ278488 | Brazil, Santa Catarina (SC) |
| H66 | S53 | C | PQ278489 | Brazil, Santa Catarina (SC) |
| H67 | S54, SC106 | C | PQ278490 | Brazil, Santa Catarina (SC) |
| H68 | S55, SC104 | C | PQ278491 | Brazil, Santa Catarina (SC) |
| H69 | S56 | C | PQ278492 | Brazil, Santa Catarina (SC) |
| H70 | S57 | C | PQ278493 | Brazil, Santa Catarina (SC) |
| H71 | S58 | C | PQ278494 | Brazil, Santa Catarina (SC) |
| H72 | S59 | C | PQ278495 | Brazil, Santa Catarina (SC) |
| H73 | S60, S72, S95, S105 | C | PQ278496 | Brazil, Santa Catarina (SC) |
| H74 | S61 | C | PQ278497 | Brazil, Santa Catarina (SC) |
| H75 | S62 | C | PQ278498 | Brazil, Santa Catarina (SC) |
| H76 | S64 | C | PQ278499 | Brazil, Santa Catarina (SC) |
| H77 | S65 | C | PQ278500 | Brazil, Santa Catarina (SC) |
| H78 | S66 | C | PQ278501 | Brazil, Santa Catarina (SC) |
| H79 | S67 | C | PQ278502 | Brazil, Santa Catarina (SC) |
| H80 | S68 | C | PQ278503 | Brazil, Santa Catarina (SC) |
| H81 | S69 | C | PQ278504 | Brazil, Santa Catarina (SC) |
| H82 | S70, S88 | C | PQ278505 | Brazil, Santa Catarina (SC) |
| H83 | S73, S97, S98 | C | PQ278506 | Brazil, Santa Catarina (SC) |
| H84 | S76 | B | PQ278507 | Brazil, Rio de Janeiro (RJ) |
| H85 | S77 | C | PQ278508 | Brazil, Santa Catarina (SC) |
| H86 | S78, S86, S101 | C | PQ278509 | Brazil, Santa Catarina (SC) |
| H87 | S80 | C | PQ278510 | Brazil, Santa Catarina (SC) |
| H88 | S81 | C | PQ278511 | Brazil, Santa Catarina (SC) |
| H89 | S83 | C | PQ278512 | Brazil, Santa Catarina (SC) |
| H90 | S85 | C | PQ278513 | Brazil, Santa Catarina (SC) |
| H91 | S91 | C | PQ278514 | Brazil, Santa Catarina (SC) |
| H92 | S92 | C | PQ278515 | Brazil, Santa Catarina (SC) |
| H93 | S93 | C | PQ278516 | Brazil, Santa Catarina (SC) |
| H94 | S96 | C | PQ278517 | Brazil, Santa Catarina (SC) |
| H95 | S99 | C | PQ278518 | Brazil, Santa Catarina (SC) |
| H96 | S100 | C | PQ278519 | Brazil, Santa Catarina (SC) |
| H97 | S102 | C | PQ278520 | Brazil, Santa Catarina (SC) |
| H98 | S103 | C | PQ278521 | Brazil, Santa Catarina (SC) |
| H99 | S107 | C | PQ278522 | Brazil, Santa Catarina (SC) |
| H100 | S108 | C | PQ278523 | Brazil, Santa Catarina (SC) |
| H101 | S109 | C | PQ278524 | Brazil, Santa Catarina (SC) |
| H102 | PG1, 2guariba, 3guariba | C | PQ278525 | Argentina, Misiones (ARG) |
| H103 | X4 | C | PQ278526 | Brazil, Rio Grande do Sul (RS) |
| H104 | X5 | C | PQ278527 | Brazil, Rio Grande do Sul (RS) |
| H105 | X9 | C | PQ278528 | Brazil, Rio Grande do Sul (RS) |
| H106 | Bede | C | PQ278529 | Brazil, Rio Grande do Sul (RS) |
| H107 | 3a_ind, 12a_AG, 20a_AG, 27a_AG, 18aa_AG, 16a_AG, 5a_AG, 33a | C | PQ278530 | Brazil, Paraná (PR) |
| H108 | 56_ind, 1_ind, 19_ind, 57_AG, 46_AG, 59_AG | C | PQ278531 | Brazil, Rio Grande do Sul (RS) |
| H109 | 13_ind, g8_17_ind, g10_24_ind, g8_16_ind, g8_18_ind, g5_10_ind, g10_23_ind, g6_12_AG, 8_AG, 60_AG, 58_AG, 9_AG, g4_6_AG, g4_7_AG, g4_8_AG, g4_9_AG | C | PQ278532 | Brazil, Rio Grande do Sul (RS) |
| H110 | 133_AG | C | PQ278533 | Brazil, Rio Grande do Sul (RS) |
| H111 | 131_AG | C | PQ278534 | Brazil, Rio Grande do Sul (RS) |
| H112 | g13_31_AG_RS g13_30_AG | C | PQ278535 | Brazil, Rio Grande do Sul (RS) |
| H113 | 137_AG_RS 135_AG | C | PQ278536 | Brazil, Rio Grande do Sul (RS) |
| H114 | 130_AG_RS 128_AG | C | PQ278537 | Brazil, Rio Grande do Sul (RS) |
| H115 | 114_AG | C | PQ278538 | Brazil, Rio Grande do Sul (RS) |
| H116 | 2_AG | C | PQ278539 | Brazil, Rio Grande do Sul (RS) |
| H117 | 24a_AG | C | PQ278540 | Brazil, Paraná (PR) |
